# Harnessing nanotechnology for stem-cell therapies: revolutionizing neurodegenerative disorder treatments – a state-of-the-art update

**DOI:** 10.3389/fphar.2025.1630475

**Published:** 2025-07-23

**Authors:** Neevashini Chengebroyen, Anmol Seelan, Kamal Yoonus Thajudeen, Saad Ali Alshehri, Aritra Biswas, Israrahmed Adur, Vino Sundararajan, Sajitha Lulu Sudhakaran, Harpreet Singh

**Affiliations:** ^1^ Jeffrey Cheah School of Medicine and Health Sciences, Monash University Malaysia, Selangor, Malaysia; ^2^ Department of Biological Sciences, Sunandan Divatia School of Science, Narsee Monjee Institute of Management Studies (NMIMS), Mumbai, India; ^3^ Department of Pharmacognosy, College of Pharmacy, King Khalid University, Abha, Saudi Arabia; ^4^ Department of Biotechnology, UNESCO Regional Centre for Biotechnology, Government of India, Faridabad, Haryana, India; ^5^ Manipal Institute of Regenerative Medicine (MIRM), Manipal Academy of Higher Education (MAHE), Bangalore, Karnataka, India; ^6^ Integrative Multiomics Lab, School of Bio-Sciences and Technology, Vellore Institute of Technology, Vellore, Tamil Nadu, India; ^7^ School of Pharmaceutical Sciences (Faculty of Pharmacy), IFTM University, Moradabad, Uttar Pradesh, India

**Keywords:** nanomaterial-conjugated regenerative therapy, neurodegenerative disorders, nanomedicine, scaffold, neuroprotective-nanotechnology stem-cell therapy, nanotechnology

## Abstract

Neurodegenerative disorders, marked by the gradual degeneration and dysfunction of neurons, pose substantial clinical challenges due to the paucity of effective therapeutic strategies and the intricate and multifactorial nature of their underlying pathophysiology. On the other hand nanotechnology, Recent advancements in nanotechnology-driven interventions have significantly augmented the therapeutic potential of stem-cell therapies for the treatment of these complex conditions. Critical limitations in current therapeutic approaches have been highlighted, while potential future directions for their therapy have been outlined. Stem cell types—embryonic, induced pluripotent, and adult neural stem cells—are categorized, with a focus on their unique biological properties and therapeutic potentials in addressing neurodegenerative conditions. The role of nanomaterials in augmenting stem cell generation, scaffold fabrication, and targeted delivery mechanisms is examined, with particular emphasis on the capacity of nanotechnology to enhance regenerative processes and neuroprotective interventions. Nanomaterial-conjugated stem cell therapies are specifically addressed, focusing on their applications in neuronal recovery and treatment monitoring. Challenges associated with stem cell therapies, including ethical considerations, immunogenicity, and the necessity for stringent clinical validation, are critically examined. The integration of nanomedicine with stem cell research is proposed as a promising strategy to overcome these challenges and facilitate the development of novel therapeutic approaches. A comprehensive framework for future research is proposed, focusing on the synergistic integration of nanotechnological advancements with stem cell therapies to improve clinical outcomes and drive innovation in the treatment of neurodegenerative disorders. By integrating existing knowledge and highlighting critical gaps, this review seeks to foster continued research and interdisciplinary collaboration, accelerating progress in this rapidly evolving field.

## Graphical Abstract

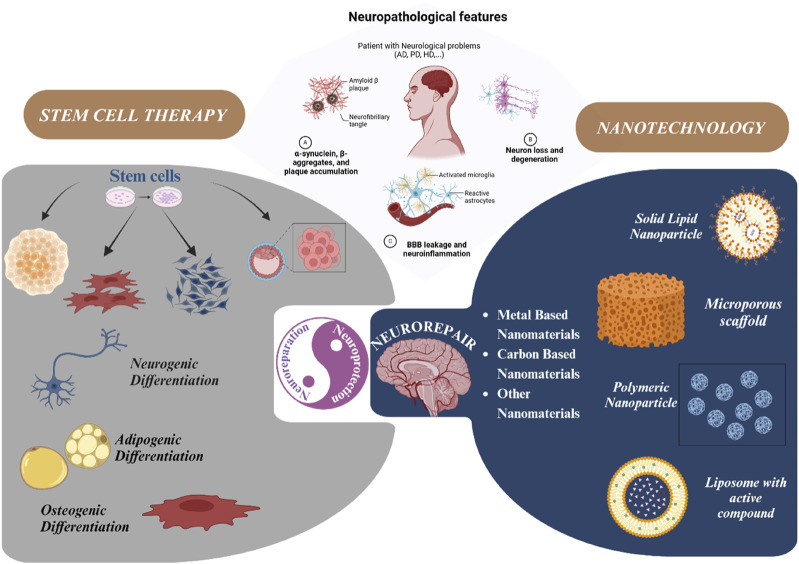

## Highlights

• The review explores how nanomaterials enhance stem-cell therapy efficacy in neurodegenerative disease treatments.
• It highlights the therapeutic potentials of various stem cells, including embryonic, induced pluripotent, and adult neural stem cells.
• Focus is placed on how nanomaterials improve stem-cell generation, scaffolding, and delivery for neuronal recovery and therapy monitoring.
• The review outlines future research needed to integrate nanotechnology with stem-cell therapies for better clinical outcomes in neurodegenerative disorders.

## 1 Introduction

Neurodegenerative disorders (NDDs) are a heterogeneous group of debilitating neurological conditions that adversely affect the lives of millions of people globally and are characterized by the progressive degeneration of nerve cells in the central nervous system (CNS) or peripheral nervous system (PNS) (Gadhave et al., 2024). Impaired neural networks and neuronal attrition hinder the ability of efficient self-renewal due to their terminally differentiated state, leading to the breakdown of fundamental communicative circuitry, culminating in clinical manifestations characterized by impaired memory, cognition, motor, and/or sensory capabilities (Wilson et al., 2023). The etiological burden of mortality and morbidity due to NDDs is increasingly posing a public health challenge, with up to one billion people suffering worldwide from these neurological disorders, and over one in three people is affected by these conditions (Vashist et al., 2023). In recent years, the absolute estimated prevalence of mortality and morbidity has increased by 39% and 15%, respectively (Ward and Goldie, 2024). The loss of quality of life, lack of effective treatment, and high cost of care affects over 63 million people with disability-adjusted life years (DALYs) of these Neurodegenerative disorders in Southeast Asia (Wang Y. et al., 2023).

Today, NDDs are still collectively the leading cause of disability and the second highest cause of death globally, serving as an urgent call to action to scale up targeted therapeutic interventions in an attempt to mitigate neurodegenerative processes (Sakowski et al., 2024). Currently, although NDDs are not curable, conventional treatments mainly alleviate cognitive manifestations and symptoms and slow down the progression of the disease (Wilson et al., 2023). Classes of drugs that are currently marketed for the treatment of NDDs include cholinesterase inhibitors, dopaminergic agents, antipsychotics, antispasmodics, and nonsteroidal anti-inflammatory drugs (NSAIDs) (Martínez-Iglesias et al., 2023). The complexity of the mechanisms associated with neuronal impairment and contradicting hypotheses regarding the physiological causes of these disorders significantly hinders the comprehension of the pathogenic processes and consequential development of effective treatments (Zhang et al., 2023). Additionally, the difficulty in targeting neuronal cell death along with the lack of robust regenerative capacity of the central nervous system (CNS) renders most currently available treatment options ineffective and insufficient (Knox et al., 2022). The variations in the specific hallmarks of the disease mechanisms and the enormous limitations in shuttling these drugs across the blood-brain barrier (BBB) further exacerbate the challenges faced in treating these diseases (Knox et al., 2022). On the other hand, nanotechnology an emerging field of interdisciplinary science that has effectively excelled in cell isolation, targeted delivery, and tracking at nano scale, has gained interest recently. The nanoscale organic and inorganic particle has certain chemical, mechanical and optical properties that are exploited to precisely control the behavior of stem cells (Jain et al., 2025). Owing to the advent of nanotechnology and pharmaceutical sciences, there are significant strides in drug delivery, formulation, and therapeutic approaches for diseases (Wang Z. et al., 2023). Combining stem cell therapy with nanotechnological advances is a major avenue for research breakthroughs to overcome the hurdles in theragnostic of neurodegenerative disorders.

Stem cell therapy, also known as regenerative therapy, focuses on stem cell usage or their derivatives to improve the repair response of impaired or damaged tissues, with strategies involving cellular replacement, regeneration of neural tissues, stabilization of neuronal networks, and alleviation of neurodegeneration at different neuronal circuitry levels (Sivandzade and Cucullo, 2021a). Nano-technological advancements in stem cell therapy associated with NDDs hold great promise for enhancing targeted stem cell delivery, protecting the survival of transplanted stem cells, and improving the efficacy and safety of these therapies (Wang Z. et al., 2023). Thus, this article potentially tends to expatiate the translational integration of nanotechnology with stem cell therapy for neurodegenerative disorders. It encompasses an exploration of stem cell classifications and their varying therapeutic potentials, along with an investigation of the pharmacological and therapeutic attributes of nanomaterials utilized in conjunction with stem cell therapy.

## 2 Neurodegenerative disorders (NDDs): gaps and prospects

A major significant global concern that arises annually is the etiological burden of mortalities and morbidities related to NDDs. Adult-onset progressive disorders that impair the function and plasticity of neuronal networks include Alzheimer’s disease (AD), Parkinson’s disease (PD), Amyotrophic lateral sclerosis (ALS), Huntington’s disease (HD) and others (Zhou and Kihara, 2023). Today, with the viewpoint of an increasingly aging society, the sociomedical burden of the prevalence, incidence, people with DALYs of NDDs propagate intensively, thus deteriorating quality of life (Feigin et al., 2020). NDDs are clinically unmanageable owing to the annual incline in aging population (Hou et al., 2019).

**FIGURE 1:**
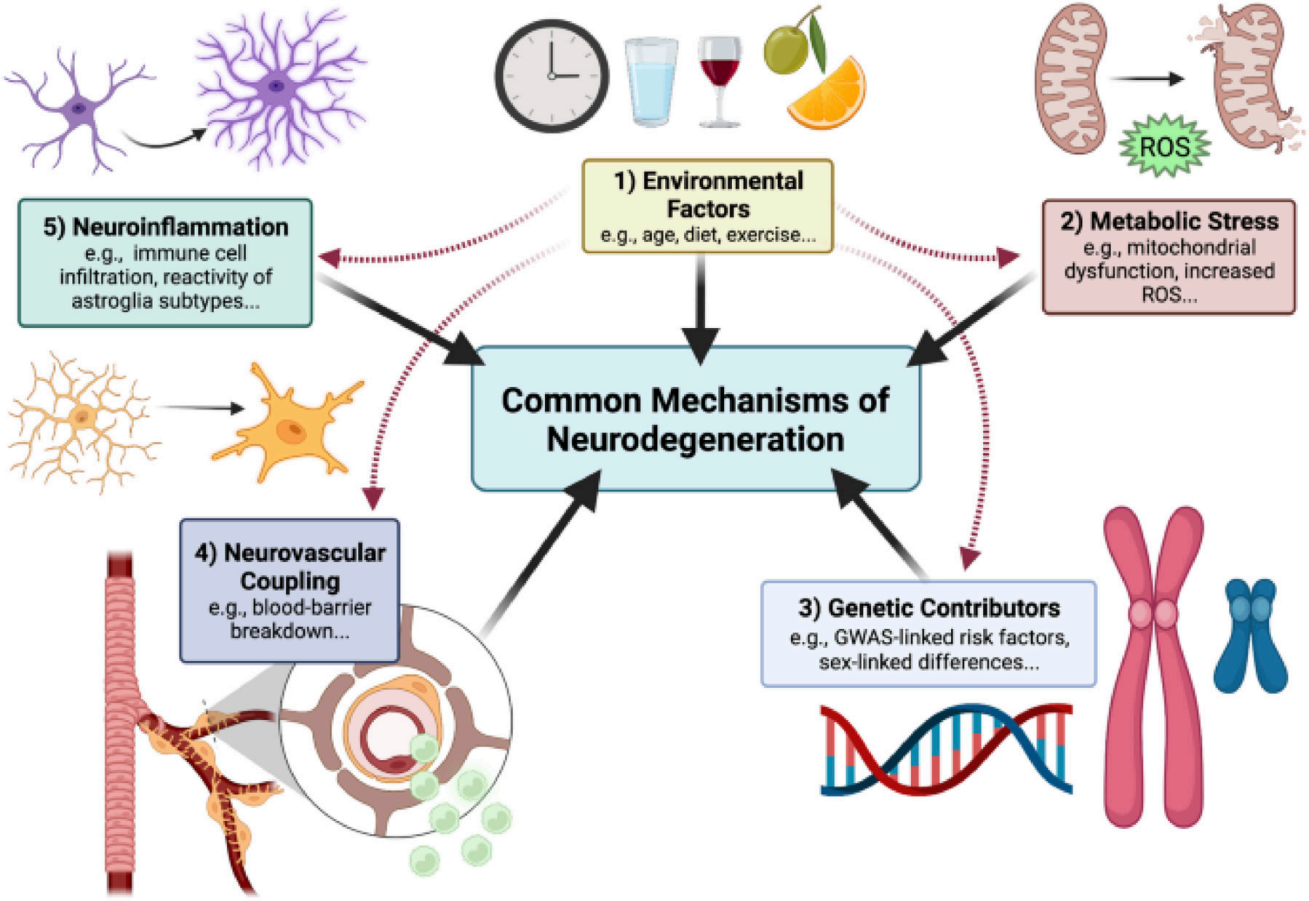
Outlined Mechanism of Neurodegenerative disease progression. Neurodegeneration is a result of misfolded protein accumulation due to genetic contributors, environmental factors, metabolic stress causing neuroinflammation and death of neuronal cells, reproduced with permission from ref. (Wareham et al., 2022), copyright 2022, BMC Springer Nature.

NDDs occur when misfolded proteins like tau, α-synuclein, amyloid-β accumulates due to environmental exposures, oxidative stress, mitochondrial dysfunction. Researchers are identifying the underlying causes and mechanism of NDDs as shown in Figure 1 for early detection and diagnosis. When it is undetected at early stage, it amplifies causing neuroinflammation and metabolic stress where cells like microglia release pro inflammatory cytokines (TNF-α, IL-1β), astrocytes disrupt neurovascular coupling and energy crisis occurs due to mitochondrial failure. The complex mechanism of neurodegeneration involves CNS along with PNS and peripheral immune system cells which contribute to BBB breakdown, synaptic dysfunction, dendritic pruning leading to exposure of tissues to toxic insults. Once the disease progresses to terminal stage it leads to neuronal cell death, cognitive and functional declines with persistent inflammation (Wareham et al., 2022).

**FIGURE 2:**
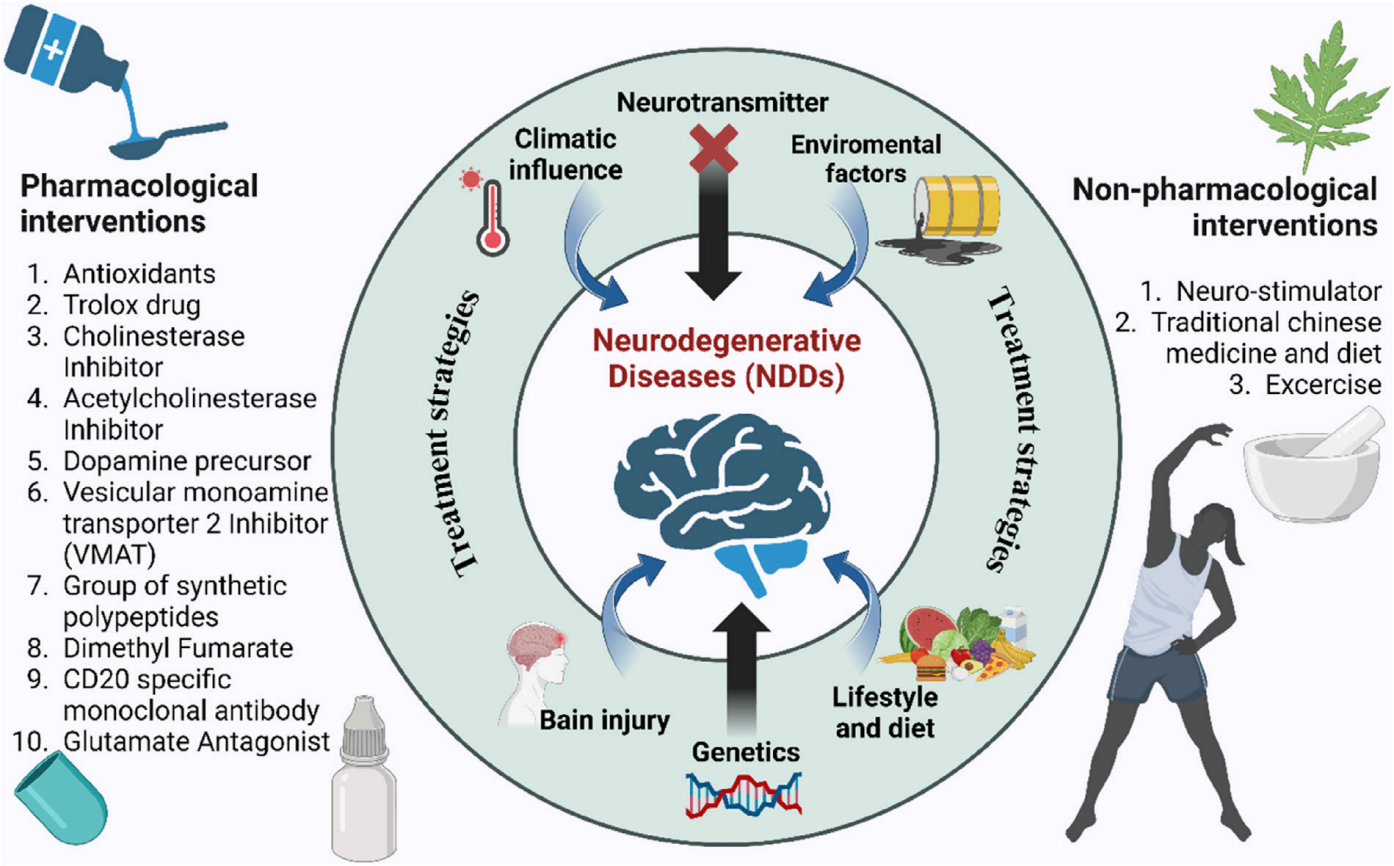
Causes and conventional treatment strategies for NDDs. Central factors include genetics, brain injury, diet, and environmental influences. Pharmacological (left) and non-pharmacological (right) interventions aim to mitigate disease progression.

Traditional treatment options are shown partially effective only in mitigating symptoms of NDDs, but no reversing the degeneration of the neuronal impairment in NDDs. With the incurable nature of NDDs, conventional medications only aim to alleviate the symptoms, marginally improving life expectancy with severe side effects which shows the deficits of conventional therapy for NDDs as the latter has no ability to replace, protect and regenerate lost neurons (Durães et al., 2018). Moreover, people with NDDs gradually become physically dependent for performing basic activities, thus rendering their life miserable and unmanageable. Economically, managing NDDs is a great burden to the patients, society and the affected families, particularly in low-income and middle-income countries (LMICs) (Feigin et al., 2020). This increasingly large burden of NDDs including high incidence and prevalence, driven by a worldwide population growth and ageing suggests that advances in prevention and management of these disorders with conventional therapies have proven to be insufficiently effective to counterbalance this ongoing increase in the absolute numbers of people affected by NDDs, estimated by Global burden of diseases, injuries, and Risk Factors study (GBD) (Feigin et al., 2020). Current conventional therapeutic approaches for NDDs are often disease-specific with different drug classes, with either aim to target the disease pathogenesis or attempt to enhance the symptoms experienced depicted in Figure 2. As instances, cholinesterase inhibitors being the first-line medications administered for AD still show limited efficacy despite new cholinesterase inhibitor drugs recently approved for treatment. Table 1 lists the conventional therapeutic regimens used in managing NDDs highlighting their mechanisms of action in targeting subtypes of NDDs along with side effects associated with their use. With being exorbitant in pricing, those conventional drugs, labeled as symptomatic therapeutic options only manage to achieve a modest improvement in NDDs’ patients’ cognitive ability rather than addressing and altering the pathology of those neurological disorders.

**TABLE 1:** Tabular representation of some conventional therapeutic regimens used in managing NDDs along with their atrocious side effects.

Name of the compound	Class of drugs	Diseases	Mechanism of action	Side effects	References
Rivastigmine (Exelon)	Cholinesterase Inhibitor	AD	• Carbamate derivative which binds reversibly and inactivates acetylcholinesterase • Causes increased concentration of acetylcholine at cholinergic synapses	Seizures, Allergic reactions, Diarrhea, Loss of strength, Irregular/slow, Heartbeat, Mental depression	Birks and Grimley Evans (2015)
Donepezil (Aricept)	Acetylcholinesterase Inhibitor	AD	• Selective and reversible inhibition of acetylcholinesterase (Ach) enzyme into breaking down to acetylcholine • Enzyme inhibition which improves cholinergic transmission	Anorexia, Insomnia, Blurred vision/Cataract, Constipation, Black, tarry stools, Breathing difficulties/shortness, Speech difficulties	Birks and Harvey (2018)
Galantamine (Razadyne)	Cholinesterase Inhibitor	AD	Binds to the Nicotinic acetylcholine receptors (nAChR) at the allosteric site, which leads to an increased Ach release and increased activity of glutaminergic neurons	Irregular Heartbeat, Blistering, peeling or loosening of the skin, Chest tightness, Hearing loss, Palpitations, Sinus Bradycardia	Olin and Schneider (2002)
Levodopa	Dopamine precursor	PD	• Prodrug of DA with various routes crosses the BBB. • Decarboxylated to form DA and for DA receptors	Dyskinesia, Ventricular arrhythmia, Sudden cardiac arrest, Hypotension, Upper respiratory tract infection	Tambasco et al. (2018)
Carbidopa	Dopa Decarboxylase	PD	• Used in combination with levodopa and inhibits the peripheral metabolism of levodopa • Helps to biosynthesize L-tryptophan to serotonin and modify L-Dopa to dopamine	Tremors, Depression Liver abnormality, Myocardial Infarction, Phlebitis	Muranova (2024)
Deutetrabenazine (Austedo)	Vesicular monoamine transporter 2 Inhibitor (VMAT)	HD	Reversibly reduces the levels of monoamines, such as DA, serotonin from nerve terminals through its active metabolites	Heart problems, Depression, Electrolyte imbalance, Hepatic disease	Frank et al. (2022)
Tetrabenazine (Xenazine)	Vesicular monoamine transporter 2 Inhibitor (VMAT)	HD	Functions within the basal ganglia which facilitate the depletion of monoamine neurotransmitters such as serotonin and DA from their storage sites. Reduces uptake into synaptic vesicles	Breath shortness, Ataxia, Insomnia, Dysphagia, Pneumonia, Bradycardia, Hypothermia	Kumar et al. (2023)
Valbenazine (Ingrezza)	Vesicular Monoamine transporter 2 Inhibitor (VMAT)	HD	Mediate the reversible inhibition of VMAT2, a transporter responsible for regulating the uptake of monoamine uptake from the cytoplasm to synaptic vesicles for storage and release	Allergic reactions Tremors Kidney disease Liver disease Congestive heart failure	Furr Stimming et al. (2023)
Glatiramer (Copaxone)	Group of synthetic polypeptides	MS	Act by modifying immune processes involved in the pathogenesis Inhibits the secretion of pro-inflammatory cytokines	Breath shortness Lumps or swelling of lymph glands Blurred vision, paralysis	Babaesfahani and Patel (2024)
Dimethyl fumarate (Tecfidera)	Dimethyl Fumarate	MS	• Involves dimethyl fumarate degradation to its active metabolite, monomethyl fumarate (MMF) • Exerts its immunomodulatory effects by altering the composition and characteristics immune cells	Gastrointestinal (GI) difficulties, Angioedema, Increased of parathyroid hormone levels, Acute pancreatitis, Pancytopenia, Hepatic injury	Ferreira et al. (2021)
Ocrevus (ocrelizumab)	CD20 specific monoclonal antibody	MS	Targets CD20, which are expressed on the surface of different types of B-cells which normally contribute to MS through activating harmful immune response	Nasopharyngitis, Respiratory tract infection, Herpes virus associated infections, breast cancer	Lamb (2022)
Riluzole	Glutamate Antagonist	ALS	Inhibitory effect on glutamate release, promotes inactivation of voltage-dependent sodium channels, thus may protect motor neurons damage and extends survival	Hepatic Complications such as acute hepatitis and icteric toxic hepatitis, Neutropenia, Tachycardia, hypertension, pancreatitis	Miller and Moore (2012)
Nuedexta (dextromethorphan hydrobromide/quinidine sulfate)	Combination of dextromethorphan hydrobromide/quinidine sulfate	ALS	Regulate excitatory neurotransmissions through sigma-1 receptor	Heart failure, Liver failure Lupus-like symptoms (patchy skin colour, muscle or joint pain)	Smith et al. (2017)
Radicava	Free radical scavenger	ALS	Suppress the generation of hydroxyl radicals and peroxynitrite radicals, hence mediate therapeutic effects with ALS -related complications	Dermatological issues such as eczema, erythema, Respiratory failure/hypoxia, Glycosuria	Cho and Shukla (2021)

Aside from the incomplete comprehensive understanding of the mechanistic complexity of Neurodegenerative disorders in humans, traditional FDA-approved drugs often fail to cross the blood-brain barrier (BBB), stymieing the potential effective treatment (Wu et al., 2021). The BBB is a major hurdle in managing NDDs with conventional therapies, as macromolecules with a molecular weight above 400 Da struggle to penetrate the brain endothelium compared to smaller molecules freely crossing the BBB through passive diffusion (Wu et al., 2021). NDDs occurs within the brain as it lies behind the BBB and endothelial cells in the brain strongly linked to continuous barrier through tight inter-endothelial junctions, further surrounded by the basal membranes, astrocytes and pericytes limit access to drug molecules from blood to brain (Knox et al., 2022). Successes from surgeries and highly invasive techniques have raised major concerns about their long-term benefit, owing to the potential damage to the blood-brain barrier as the barrier functions essentially in preventing the brain from circulating pathogens, toxins and aid in brain homeostasis, thus impeding quality of life post treatment (Sweeney et al., 2018). Thus, the advanced nature of BBB, coupled with poor permeative drug potency accounts for the absence of effective current options for NDDs (Knox et al., 2022). Limitations caused by BBB, disadvantages of conventional drug usage owing to severe side effects, as mentioned above have led to the unmet need for novel therapeutic approaches for NDDs. Out of these approaches employed, stem cell therapy and nanotechnological advances are safe and promising platforms which are currently emerging for targeted therapeutic approaches for the treatment of NDDs.

After profound investigation about neurodegenerative disorders and employment of various therapeutic strategies targeting permanent cures, stem cell (SC) therapy is one approach which showcased significant potential for treatment of a wide range of diseases, thus seconding the high success potential of stem cell usage for treating and curing NDDs (Nie et al., 2023). Providing hope for many patients, SC with their self-renewal and pluripotent properties makes them suitable candidates for cell transplantation/therapy. Another approach to mitigate the limitations of current therapies has emerged as Nanotechnology (Abdul et al., 2024). This technology, employing materials in nanoscale (1–1,000 nm) capable of interacting with biological systems at molecular level, enhances brain-drug delivery for NDDs stem cell therapy (Dash et al., 2022). Stem cell nanotechnology, being a novel field, has yielded several successes from experimental studies conducted to establish the significance of nanostructures, nanotechnology and nanomaterials in the development of stem-cell based treatment for NDDs. There are promising findings which provide strong proof that application of nanotechnology in SC therapy has the ability to alter the pathology of NDDs owing to the unique properties of nanomaterials which make them appropriate the address the barriers in stem-cell therapy (Dash et al., 2022). Recent major diagnostic and therapeutic advancements of nanotechnological interventions associated with SC therapy have shown great prospects of this marriage which continue to overcome the burden of NDDs intending to provide permanent cure (Oz et al., 2023).

## 3 Overview of stem cells and categorization of stem cells

**FIGURE 3:**
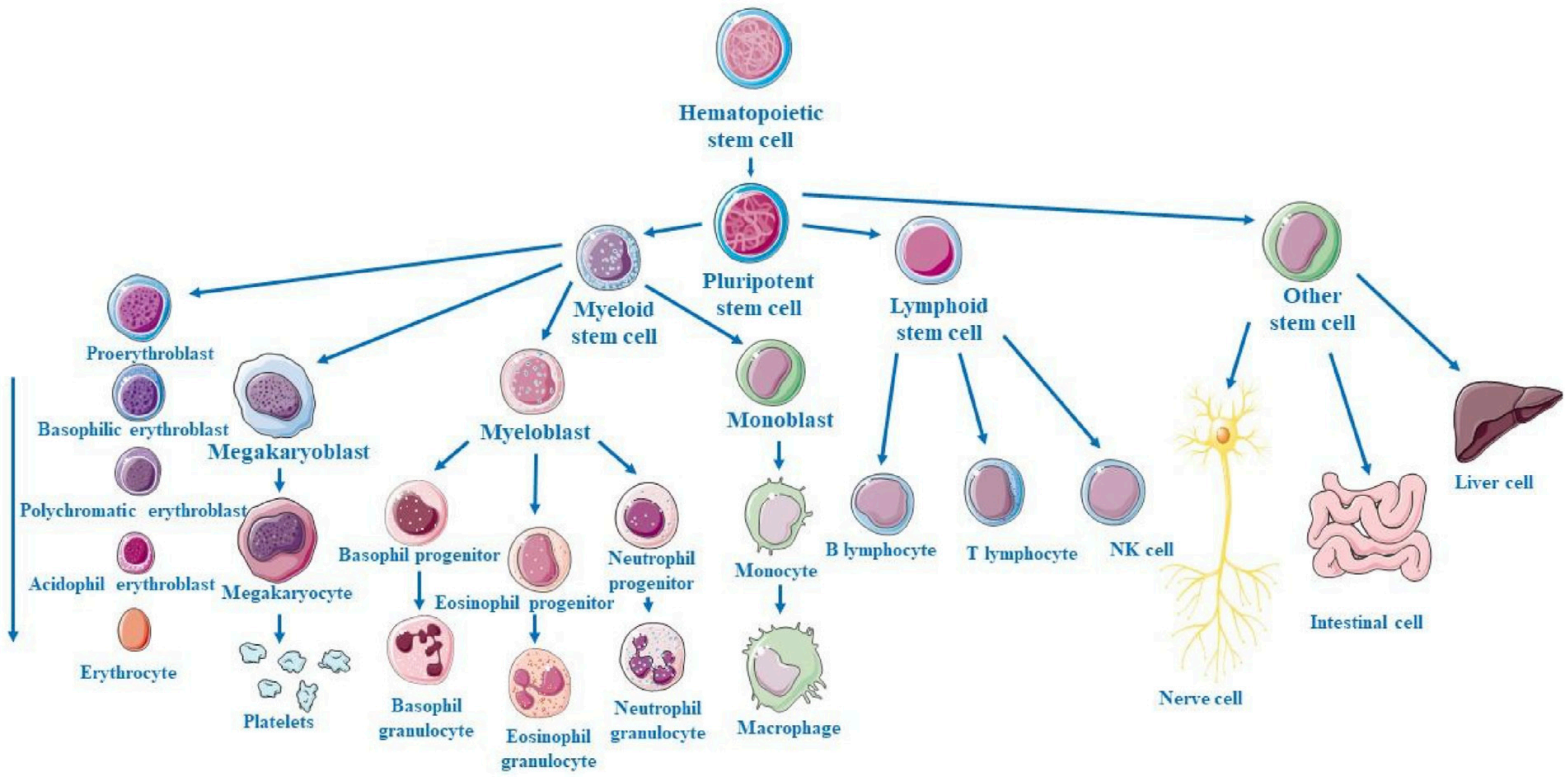
Schematic representation of hematopoietic stem cell (HSC) progression from multipotent HSCs to common progenitors, differentiation into various blood cell lineages of distinct myeloid and lymphoid cell types.

Stem cells are a unique population of cells which are characterized by their distinct capabilities to self-renewal by cell division and differentiation into an extensive variety of multiple cell lineages (Ahani-Nahayati et al., 2021). Present in the human body, these unspecialized and self-renewing cells are key mediators in the development of neonates and restorative processes after injury or diseases and possessing the ability of self-replication through asymmetric cell division, they pose as the pivotal source from which specialized cell types within differentiated tissues and organs are derived (Beshiri et al., 2024). Within the neonate stage of life, stem cells proliferate into multiple cell lineages and cell types which enables continued development and growth to mature into an adult, while in adults, their primary role is inclined towards a regenerative and restoration nature on a cellular level (Ceja et al., 2023). Multipotent stem cells called hematopoietic stem cells (HSCs) are located in the bone marrow and are responsible for the process of hematopoiesis, which produces all blood cells as illustrated in Figure 3. Myeloid and lymphoid progenitors are the two primary progenitor cell types that HSCs differentiate into. Myeloid progenitors go on to differentiate into platelets (thrombocytes), which aid in blood coagulation, red blood cells (erythrocytes), which carry oxygen, and different kinds of white blood cells (leukocytes), such as neutrophils, eosinophils, basophils, monocytes, and macrophages, which are involved in inflammation and immune response. Lymphoid progenitors give rise to natural killer (NK) cells, which are a component of the innate immune system, as well as T and B cells, which are essential for adaptive immunity. The continual supply of varied blood cells required for oxygen transport, the immune system, and clotting is ensured by this hierarchical differentiation (Mikkola and Orkin, 2006). Mesenchymal stem cells (MSCs) are multipotent stromal cells capable of differentiating into various cell types, contributing to the regeneration and repair of various tissues. Primarily, MSCs have the capacity to differentiate into osteoblasts (bone-forming cells), chondrocytes (cartilage-forming cells), and adipocytes (fat cells). Additionally, under certain conditions, MSCs can also give rise to myocytes (muscle cells), tenocytes (tendon cells), and even neurons and other cell types found in the nervous system. This broad differentiation potential makes MSCs a valuable resource in regenerative medicine and tissue engineering, offering promising therapeutic approaches for conditions like bone and cartilage damage, cardiovascular diseases, and neurological disorders (Ding et al., 2011).

Stem cells possess distinct properties that distinguish them from terminally differentiated cells allowing for their specific physiological functions. Potency refers to the varying ability of stem cells to differentiate into multiple cell types where they are classified by their origin as well as their differential potential (Hoang et al., 2022; Zhu et al., 2024). Based on their origin, they are divided into two major categories namely embryonic stem cells (ESCs) and adult stem cells (AdSCs). Based on differential potency, there are different classifications of stem cells, including ESCs, induced pluripotent cells (iPSCs), mesenchymal stem cells (MSCs) and neural stem cells (NSCs) (Wang et al., 2024). Totipotent or omnipotent stem embryonic stem cells form embryonic tissues with a high differential potency forming multitude of cell lineages required for the developmental phase of an adult. ESCs are derived from the inner cell mass (ICM) of blastocysts whereas relative to ESCs, AdSCs has a limited differential potency and can be found in various tissues in the body, functioning to maintain tissue homeostasis. Pluripotent stem cells (PSCs) can differentiate into cells of all three germ layers except extraembryonic structures, such as placenta (Wang et al., 2024).

Although stem cells therapy is still in its infancy, stem cells began to pave their way in modern regenerative medicine in the 1950s with the first bone marrow transplantation in 1956 (Casado-Díaz, 2022). This breakthrough has shed light on the potential clinical therapeutic applications with objectives typically focused on cellular replacement or on providing environmental enrichment. The emergence of stem cell therapy in Neurodegenerative disorders was in the 1980s where its potential was examined in patients suffering from PD and they were treated with fetal mesencephalic tissue transplantation (Sivandzade and Cucullo, 2021a). It is a groundbreaking strategy in the field regenerative medicine in NDDs while each type of stem cells has its different potencies and these strategies involve translating stem cells including ESCs/iPSCs into novel NDDs therapies (Mousaei et al., 2022). Stem cells, with their homing effect can recognize, migrate to injury or lesion sites and help in generating new neural cells for reparation.

The classification of stem cells, based on their potential for cell type production and derivation methods, elucidates their distinct characteristics, unique properties, and advantages, which significantly influence the potential effect of cellular therapies on NDDS mechanisms. At present, embryonic stem cells (ESCs), neural stem cells, induced pluripotent stem cells (iPSCs) and mesenchymal stem cells (MSCs) are prolific stem cells used in the NDDs therapeutical strategies and each stem cells have their own advantages and limitations which thus require understanding of their origin, differential potency, distinct characteristics and their rational design on applications and outcome.

### 3.1 Embryonic stem cells (ESCs)

ESCs are pluripotent with the ability to self-renew indefinitely with a high differentiation potency into almost all cell types of the CNS. These cells, derived from inner cell mass of blastocyst, may be expanded *in vitro* for extended years while their differentiation capacity into multitude of specialized cell types is retained. Consequently, they serve not only as a valuable model for studying and understanding early mammalian embryonic development, but also a pivotal source for generating specialized cells essential for therapeutic applications. Currently ESCs is being utilized as an inexhaustible source of neurons in numerous neurodegenerative transplantation models including experimental study involving new candidate drugs for NDDs. Approved by the Food and drug Administration (FDA) in January 2009 for first clinical application, ESCs -derived tissue was successfully used in CNS, producing oligodendrocytes for a spinal cord injury treatment (Romito and Cobellis, 2016). In 2002, Isacson utilized undifferentiated mouse ESCs to demonstrate that they could incorporate into the striatum of a rat PD model, further differentiating into glial subtypes dopaminergic (DA) neurons, thus reversing the locomotive deficit of parkinsonian rats. This experimental work in rodents led to experiments evaluating the potential of human ESCs in other similar animal experiments. Laboratory techniques were developed to efficiently differentiate ESCs *in vitro* into neuronal progenitor cells (NPCs) and neuronal and glial subtypes (Guillaume and Zhang, 2008). As an instance, neural cells are differentiated *in vitro* from ESCs, thus demonstrating broad cellular heterogeneity. Human embryonic stem cells (hESCS) -derived NPCs present a significant opportunity for directed cell differentiation with the primary objective and potential to functionally integrate into the host’s endogenous neuronal circuitry, thereby restoring functional neurological deficits. hESCs being the potential source for cell replacement therapy require intense understanding of the nervous system to successfully expose the directed differentiated cell to proper concentrations and sequence factors (Rahman et al., 2022). One priority in cell replacement therapy for Neurodegenerative disorders (NDDs) has been Parkinson’s disease (PD), where hESCs have successfully generated electrophysiologically active midbrain dopamine neurons through the strategic and systematic application of signaling molecules Fibroblast Growth Factor 8 (FGF-8) and Sonic Hedgehog (Shh), playing prerequisite roles in development and differentiation of various tissues in the nervous system (Guillaume and Zhang, 2008). In addition, motor neurons derived from ESCs have shown the ability to replace defective motor neurons in rodent models of hereditary amyotrophic lateral sclerosis (ALS), with neurological and behavioral tests confirming successful functional restoration. Moreover, it is observed that the tropic factors support ESCs differentiated into midbrain dopaminergic neuron that improve motor deficit in Parkinson’s models (Gadhave et al., 2024). For instance, ESC-derived neural progenitors are encapsulated with peptide-based metrices which slowly release neurotrophic factors (Wilson et al., 2023). With recent 3D organoid culture mouse ESCs implanted in nanofiber scaffold exhibit better dopaminergic differentiation (Vashist et al., 2023). Utilization of undifferentiated ESCs raises concerns about their potential to form tumors and teratomas due to their propensity for continual proliferation. However, inducing cell differentiation can diminish the cell multipotency, hence mitigating associated transplantation risks. Despite this, numerous ethical concerns remain regarding the use of ESCs for treatment.

### 3.2 Induced pluripotent stem cells (iPSCs)

Even though hESCs might be utilized to treat NDDs, there are constant ethical difficulties regarding the usage of human embryos along with tissue rejection post transplantation, thus giving rise to the generation of pluripotent stem cells (PSCs) directly from the recipients’ own cells in an aim to circumvent these controversies about ESCs-derived therapies (Takahashi and Yamanaka, 2006). In 2006, Yamanaka pioneered alternative therapeutic options by establishing the capacity to reprogram differentiated cells, primarily fibroblasts, into early-stage undifferentiated cells and ESCs-like cells, termed as Induced Pluripotent SCs (iPSCs). This groundbreaking discovery of iPSCs have enabled researchers to acquire PSCs without relying on embryos, thus overcoming the ethical controversies. This innovative technique enables the de-differentiation cells, challenging previously traditional held notion whose developmental fate was irrevocably determined (Karami et al., 2023). The induction of iPSCs were generated from mouse embryonic and adult fibroblast cultures by activating four murine transcription factors genes: Oct4 (also known as Pou5F1), Sox2, c-Myc and Klf4 (Takahashi and Yamanaka, 2006; Abad et al., 2013). While experimenting on reprogramming somatic cells through transfer of nuclear contents into oocytes, it has been indicated that ESCs possess these factors genes which can confer totipotency features to somatic cells, thus hypothesized that the expression of these combined transcription factor genes have a crucial capacity of reprogramming somatic cells to pluripotency (Takahashi and Yamanaka, 2006). Furthermore, with their proliferative and differentiation capabilities, iPSCs are capable of establishing developmental cell layers under empirical conditions, achieving better outcomes than ESCs-derived therapy. The application of iPSCs in research has been two-fold, serving both for human disease modelling as well as potential generation of new therapeutic techniques, particularly for intricate human diseases like NDDs. Those self-renewal pool of cells offer significant benefits over conventional animal models of neurological pathology by more precisely mirroring the human genome. As instance, with the aim of restoring dopamine levels and mitigating motor deficit in PD, iPSCs have been shown to differentiate into dopaminergic (DA) precursor cells and relocate into the substantia nigra of PD murine models, where further development of DA neurons occurred over the long term and integrate functionally into the brain parenchyma. The transplanted iPSC-derived from neural progenitors have been engineered dopaminergic progenitors survived and functioned without tumor formation in Parkinson’s model (Ward and Goldie, 2024). Similar to ESCs, iPSc contribute mechanistically via cell replacement and paracrine growth factor, immunomodulation (Wang Y. et al., 2023). A comparative study, involving five PD rhesus monkeys as recipients for autologous iPSCs-derived midbrain dopaminergic neural progenitor cells *in vivo* implantation has demonstrated that PD monkeys with autologous transplantation therapy, but not allogenic transplantation exhibited recovery and improvement from motor deficient symptoms and mood disorder symptoms (Tao et al., 2021). This survivability of autologous iPSCs -derived neurons/progenitors during *in vivo* cell transplantation has resulted into successful and accurate reinnervation, removing the need for an immunosuppressive regimen post implant for NDDs patients (Yefroyev and Jin, 2022). This pivotal feature makes iPSCs to be remarkable sources for biomedical applications, pivotal discovery for tissue engineering, thus showing great potential for NDDs SC therapy.

### 3.3 Neural adult stem cells

Neural stem cells are adult SCs with multipotency capability, allowing them to differentiate into unipotent cells of the residing tissue for regenerative purposes. The multipotency differentiates them into neurons, astrocytes and oligodendrocytes (Sakowski et al., 2024; Martínez-Iglesias et al., 2023). Adult SCs can be obtained from several various regions of adult organisms. Their immunomodulatory power of adult stem cells enhance regeneration where they can survive and migrate to the injured site, delay loss of motoneurons, interact with the proinflammatory environment to effect repair. This helper profile has largely been observed in Neural stem cells (NSCs). First described in 1960, NSCs are a class of multipotent cells defined on their basis of being a valuable resource for the management of NDDs because of their robust self-renewal capacity and differentiation potential into the three major neural lineages, including neurons, astrocytes, and oligodendrocytes (Hedayat et al., 2023). Beside their differentiation capabilities, they are known to release neurotrophic factors, proangiogenic molecules and anti-inflammatory agents that in turn support neurogenesis, angiogenesis and ameliorate neuroinflammation and oxidative stress (Masoudi et al., 2020). NSCs aided endogenous neurogenesis has been established in various pathological conditions, such as epilepsy, MS, stroke, and AD. In AD, the rebuilding of basal forebrain cholinergic neurons of transplanted NSCs form new neurons that integrate into circuit networks (Zhang et al., 2023). However, relying solely on endogenous repair mechanisms is insufficient to address the multifaceted nature of these NDDs. Consequently, NSCs transplantation emerges as a promising strategy within the realm of regenerative medicine, experiencing escalating interest and offer viable path in achieving therapeutic developments (Nie et al., 2023). A recent research study aimed to investigate the therapeutic potential of human NSCs transplantation targeted to the fimbria fornix which significantly improved cognition in an AD murine model. Animals were subjected to intracranial NSCs transplantation which further confirmed in the safety and efficacy of human NSCs in ameliorating cognitive function, further suggesting the immunomodulatory capacity of NSCs that increased microglial and amyloid phagocytosis, hence supporting potential preclinical development of NSCs -aided therapy in AD patients (McGinley et al., 2018). The immune system prod shifts microglia towards anti-inflammatory (M2) phenotypes and reduce pro-inflammatory cytokines when transplanted with NSCs during a neurodegeneration disease. While in AD model, the microglia activation reduced whereas astrocytes proliferated enhancing Aβ clearance and water flux (AQP4) in the glymphatic system, thereby reducing neuroinflammation (Knox et al., 2022). In accordance with this, the use of NSC-derived EVs has reported the neuroprotective effect for PD in a study where the levels of ROS and pro-inflammatory cytokines were reduced, which led to the protection of stem cell neurons’ loss. Regarding the treatment of HD, mesenchymal stem cells (MSCs) have already exerted an effect on reducing HD aggregation as well as enhancing endogenous neurogenesis; therefore, they are promising precursory therapeutic agents.

**FIGURE 4:**
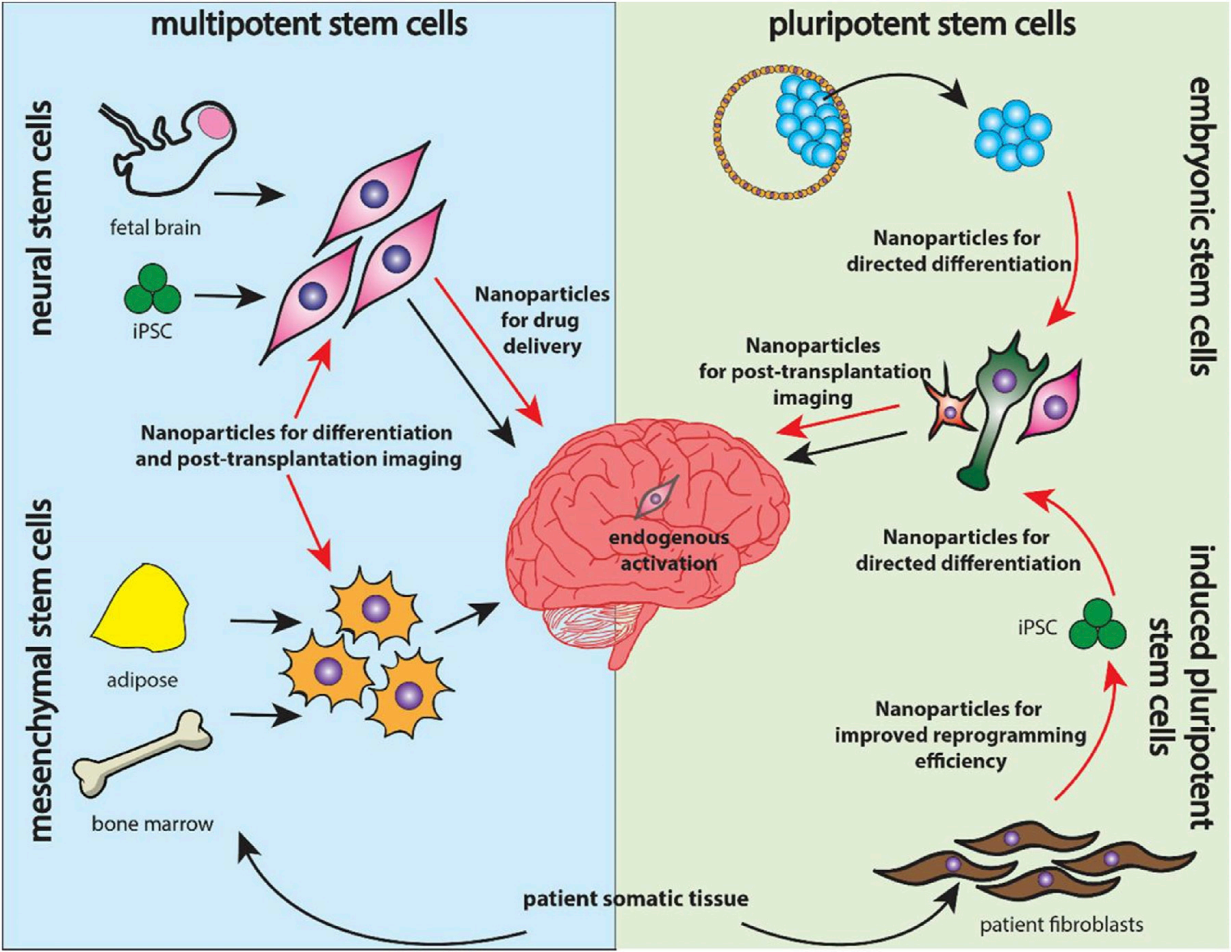
Overview of stem cell categories explored for neurodegenerative therapies. Multipotent neural and mesenchymal stem cells (left) are delivered into the brain alongside nanoparticle systems, while pluripotent embryonic and induced pluripotent stem cells (right) utilize nanoparticles for reprogramming, differentiation, and targeted drug delivery, reproduced with permission from Ref. (Vissers et al., 2019), Copyright 2019, Elsevier.

In addition, NSCs are useful in the establishment of AD and related dementias whereby they provide understanding on the intrinsic changes in neurons and spread of prion-like proteins that is important in ADMET approaches. Another demonstration of the usage of NSCs in ALS has provided positive results with regard to MSCs’ possibility to slow down the disease developing process and increase neuronal plasticity. Moreover, it has shown that NSC-derived EVs can alleviate AD related pathology such as decrease amyloid-beta plaques, decrease activation of microglia and thus, improving the cognitive ability of the 5xFAD mouse model (Victoria and Zurzolo, 2017). Moreover, the nanoparticles are designed for reprogramming and differentiating ESCs, MSCs, NSCs for the narrower applications in therapy. Importantly, it is the nanoparticle functioning and assisting in tracking, microenvironment modulation, drug delivery deciding the application of stem cells in the therapy. This is illustrated in Figure 4 where four distinct sources have been investigated for nanomaterial-enhanced regenerative therapies targeting NDDs (Vissers et al., 2019). Altogether, these studies have demonstrated the complex treatment application of NSCs and their derivatives in caring for Neurodegenerative disorders and highlighting the need for further research efforts to enhance NSCs’ method and effectiveness in treating patients with Neurodegenerative disorders to advance the present research into practice. Altogether, considering the stem cell therapies, including the NSCs, as one of the prospective lines for Neurodegenerative disorders treatment, further research and a refinement of experimental data are needed to translate these findings into clinically effective treatments (Sivandzade and Cucullo, 2021a).

## 4 Therapeutic modalities enabled by stem cell

**FIGURE 5:**
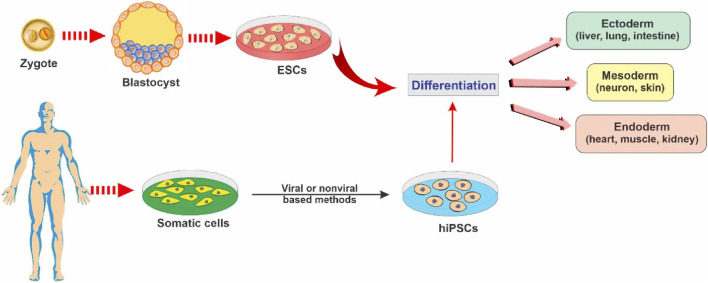
Illustrative schematic showing key stem cell types and their differentiation potential into multiple cellular lineages, reproduced with permission from ref (Sivandzade and Cucullo, 2021a). Copyright 2021, MDPI.

In contemporary biomedical research, the realm of stem cell biology has emerged as a profoundly captivating domain. The concept of regenerative medicine has undergone substantial advancement, chiefly propelled by the remarkable and nuanced attributes of stem cells, which exhibit an innate capacity for self-renewal and pluripotent differentiation into a myriad of specialized cellular lineages. To achieve a comprehensive grasp of the profound import of these exceptional cellular entities within the tapestry of modern medical practices, this section endeavors to expound upon the intricate facets that underlie the application of stem cell therapies (Mayhall et al., 2004). The fact that differentiated and degenerated organs can be reversed by inducing pluripotency in the differentiated cell line was first demonstrated by Shinya Yamanaka and his colleagues. They demonstrated that differentiated mouse cell line could be reprogrammed to form Pluripotent Stem Cells (PSCs) via four factors (Oct3/4, Sox2, c-Myc, and Klf4) which was later given the name of Yamanaka Factors (Takahashi and Yamanaka, 2006). Figure 5 demonstrates mechanistic differentiation of (ESCs) and somatic cells reprogrammed into induced pluripotent stem cells (iPSCs) toward specific cell lineages.

Stem cells possess the inherent capability to enact a paradigm shift in current therapeutic methodologies, ushering in novel avenues for addressing conditions and injuries hitherto deemed insurmountable, exemplified prominently by afflictions like spinal cord injuries (Klein and Svendsen, 2005), Type 1 diabetes (Zhao et al., 2012), Alzheimer’s as well as Cancer (Mimeault et al., 2007). Stem cell research has recently received a lot of attention because of the inherent plasticity of stem cells as well as its potential to repair tissues that were thought to be untreatable and restore function to a degenerating organ (Thapak et al., 2022). Although the research has largely been restricted to lower organisms since regeneration of entire organs or tissues are inherent properties of lower organisms. The property of regeneration is hardly present in higher mammalian lineage, instead the mammalian lineage has the property of repair which is a much different process after all. However, investigating how regeneration affects smaller creatures has provided and continue to provide us invaluable information and comprehensive ideas of the mechanisms at play in the process and open up new avenues for induction of these mechanisms in medical treatment of higher organisms (Karami et al., 2015).

### 4.1 Regenerative therapy

**FIGURE 6:**
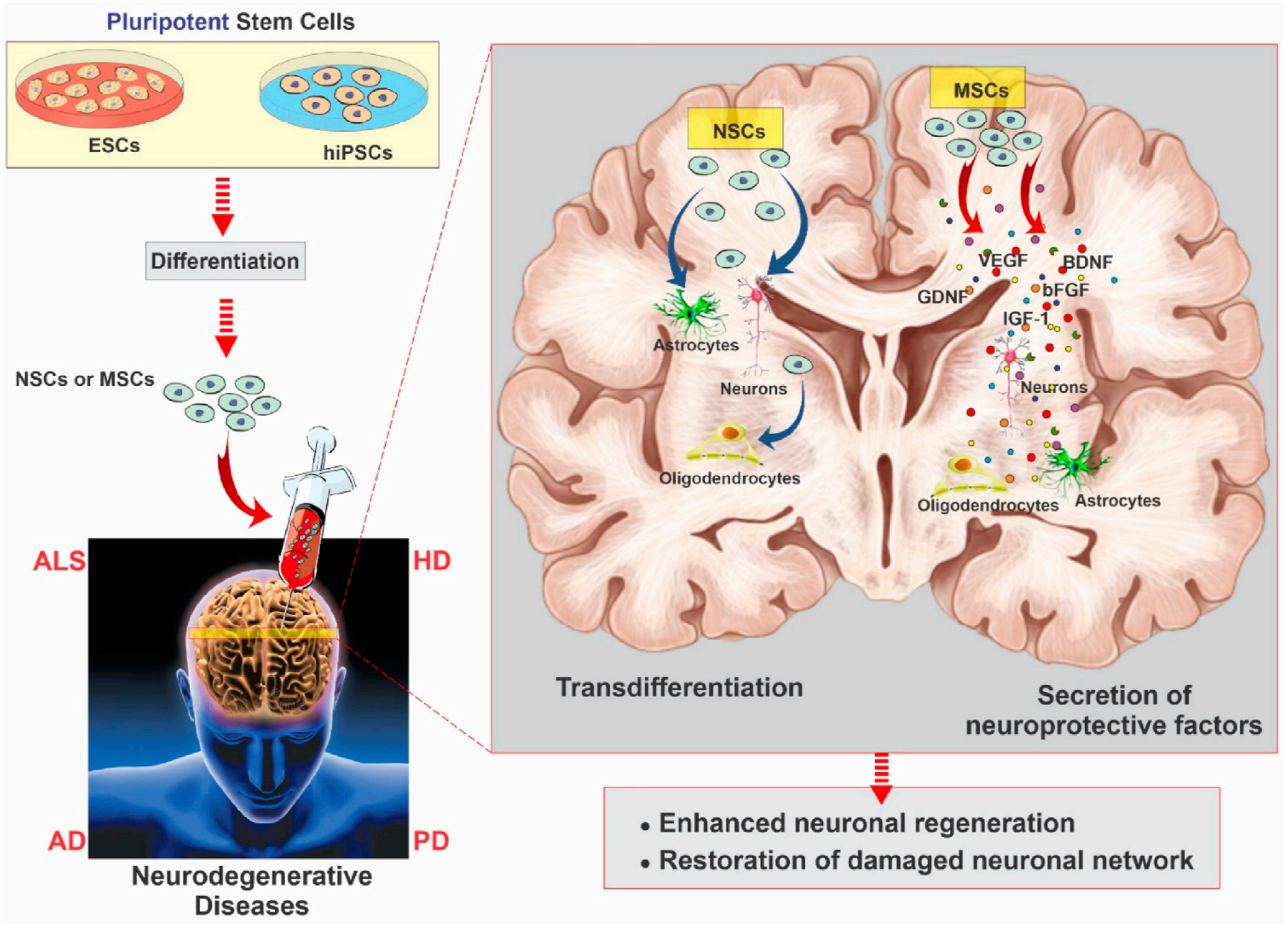
Schematic of neurodegenerative disease modeling using hiPSCs and ESCs. These cells differentiate into neuronal progenitors and MSCs, giving rise to neurons, astrocytes, oligodendrocytes, and other glial lineages. MSCs further support neural repair via secretion of growth and neurotrophic factors that promote angiogenesis, neurogenesis, and immunomodulation, reproduced with permission from ref (Sivandzade and Cucullo, 2021a). Copyright 2021, MDPI.

Regenerative medicine using stem cell-based therapy has spearheaded its efficacy in nerve regeneration treatment strategies for CNS -related brain disorders which requires significant preclinical research over the last few years. NDDs, characterized by progressive loss of structure, function or number of neuronal cells in the brain or spinal cord encompass complex associated mechanisms which present enormous limitations for a vast variety of therapeutic drugs (Sivandzade and Cucullo, 2021a). Regenerative stem cell therapy has revolutionized medicine over the years with their therapeutic applications focusing on either cellular replacement or providing environmental enrichment (Sivandzade and Cucullo, 2021a). With regard of the pivotal objectives of regenerative SC therapy which includes generating distinct neuronal subtypes and recapitulating a neural network analogous to the neuronal loss and enriching the environment for supporting host neurons through the production of neurotrophic and scavenging harmful factors or establishing auxiliary neural networks around impaired areas, researchers focus on creating a more conducive environment for therapies, aiming to enhance the success of regenerative SC therapy in NDDs patients. Owing to the exclusive multipotency capacity and distinctive assets of MSCs which enable their differentiation into a range of endodermal and ectodermal lineages, from neurons to glial cells, researchers have prioritized on human MSCs to enhance SCs-based therapeutic approaches (Sivandzade and Cucullo, 2021a). The potential of regenerative therapy in NDDs was first explored in the 1980s when fetal MSCs were transplanted in Mexican recipients suffering from PD which rendered NDDs to become a landmark in the history of regenerative stem cell therapy (Shariati et al., 2020). Additionally, MSCs with their ability to release neurotrophic factor and exhibit immunomodulatory properties have been highlighted as a novel approach for AD regenerative SC therapy. Bone marrow MSCs (BM-MSCs) and adipose MSCs (ADMSCs) have been discovered their ability to differentiate into neuronal and glial lineages apart from mesodermal lineages and this therapeutic translation has significantly demonstrated the pivotal features of MSCs with the therapeutic effects after transplantation into the brain are mediated by release of growth factors, anti-apoptotic molecules and anti-inflammatory cytokines building a favorable environment for neuronal regeneration, remyelination and enhancement of the cerebral flow in NDDs. As instance, the therapeutic effect of BM-MSCs intracerebral transplantation was evaluated in AD mice model which resulted in significant reduction of the amyloid-beta (Aβ) deposition and restoration of memory deficits as well as defective microglial function by modulation of immune/inflammatory responses, thus ameliorating the overall pathophysiology and proving to be potent therapeutic agent NDDs regeneration (Lee et al., 2009) A schematic illustrating neurodegenerative disease modeling using hiPSCs and ESCs is presented in Figure 6, adapted from Sivandzade and Cucullo, (2021a). The multiplicity benefits of SC transplantation have recently contributed in neuroprotection for AD whereby human dental pulp stem cells (hDPSCs) transplanted *in vitro* AD cell models, have demonstrated significant improvement in cognitive decline and mitigating neuropathology through AKT/GSK3β-mediated Nrf2 activation and nuclear accumulation. This corroborates the neuroprotective potential of hDPSCs to reshape the neuropathological microenvironment in AD models and alleviating the associated hallmark pathological features such as liposaccharide -induced oxidative stress and apoptosis, neurofibrillary tangles, thus posing as a highly promising therapeutic candidate for regenerative stem cell therapy for NDDs (Xiong et al., 2024). The underlying strategies for stem cell therapy in ALS follow similar objectives as described above: (a) replace deficit motor neurons, (b) establish a microenvironment. The usage of fetal-spinal-cord-derived NPCs, first approved by FDA in 2010 was aimed to assess the safety of transplantation in affected recipients. Several other stem cell research have demonstrated the efficacy and safety of intracerebral, intrathecal and intraspinal MSC transplantation in ALS patients. Owing to the unknown pathogenesis and limited understanding about ALS spreading mechanism, few conclusive results have been reported concerning SC cell transplantation thus more related undergoing trials are expected in the upcoming years. In the area of regenerative stem cell therapy much has been accomplished although there are still many challenges that still exist. These challenges include the need to improve the methods of delivery of cells to achieve the maximum level of impact, the crucial issue of maintaining the viability of the transplanted cells after transplantation and the integration of such cells into the patients’ body, and duties of tackling the ethical and legal questions related to stem cell research. In addition, a foremost need for broader and more elaborate clinical trials is necessary to confirm and prove the long-term safety and effectiveness of stem cell therapy for various neurological disorders collectively.

### 4.2 Neuroprotective therapy

**FIGURE 7:**
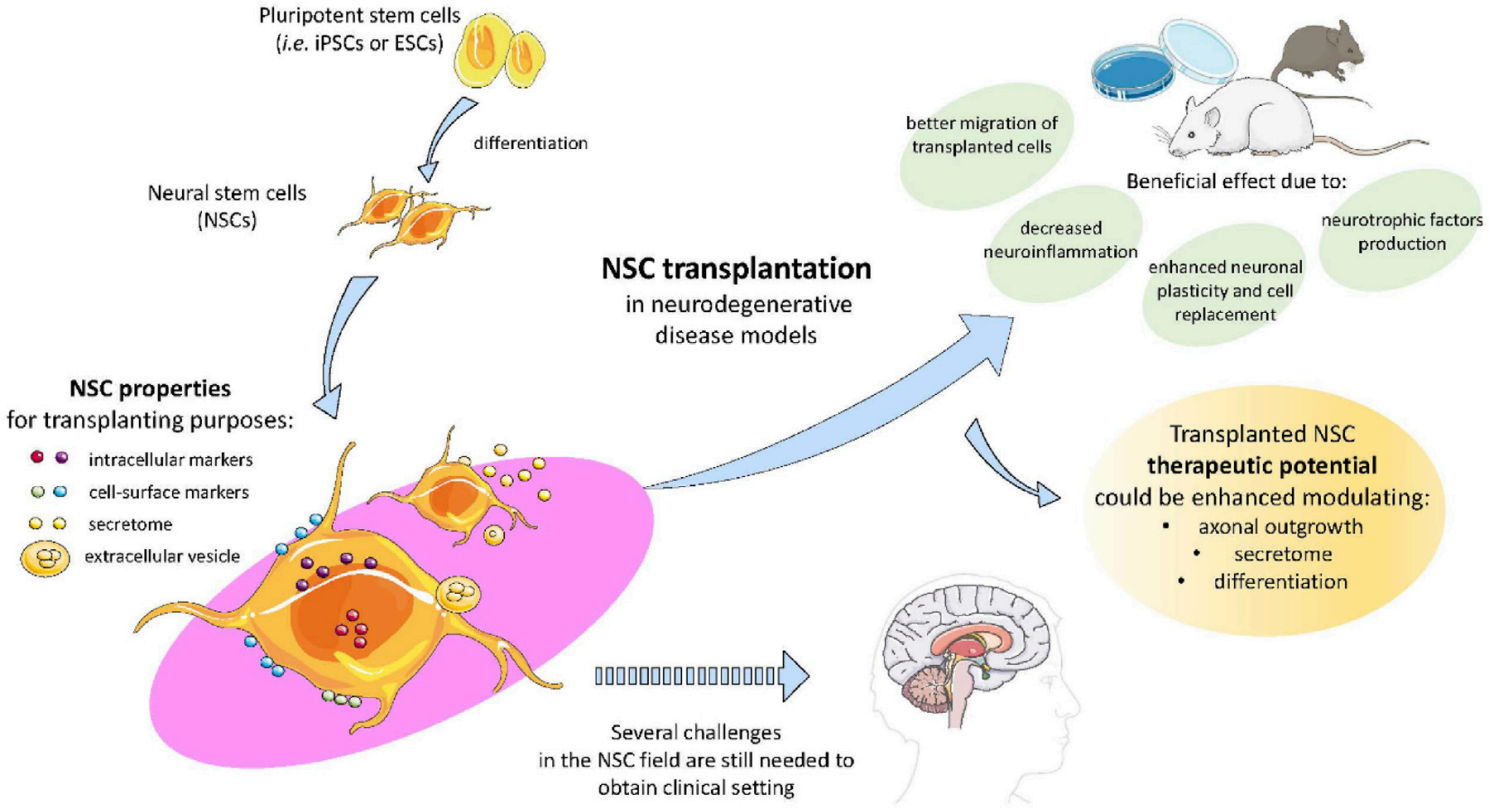
The figure illustrates the potential of pluripotent stem cells like iPScs and ESCs in therapeutic potential modulating axonal growth, secretory activity, and differentiation in NDDs. These NSCs transplantation proved to decrease neuroinflammation, enhanced neuronal plasticity and regeneration due to NSCs secretome and extracellular vesicle characteristics, reproduced with permission from ref (De Gioia et al., 2020). Copyright 2020, MDPI.

Stem cell therapy is a multifaceted approach, and one arm of this research focuses on neuroprotective therapy. Neuroprotective therapy, which strives to protect and fortify the delicate architecture of the central nervous system against the ravages of disease, injury, and degeneration, is a ray of hope in the realms of clinical medicine and neuroscience. The intricate web of synapses and neurons that make up the symphony of human cognition are susceptible to a wide range of risks, including stroke, catastrophic brain injury, and sneaky neurodegenerative illnesses like Alzheimer’s and Parkinson’s (Hung et al., 2010). Neuroprotective therapy employs a variety of pharmacological, cellular, and molecular strategies to lessen damage and preserve brain vitality. It emerges as a tactical approach in this effort. It tries to intervene at the site where dysfunction and damage meet. NSC transplantation has been proposed as a potential therapeutic strategy and appears to have a positive effect on neurodegeneration through a number of mechanisms, including the production of neurotrophic factors, decreased neuroinflammation, improved neuronal plasticity, encouraged synaptogenesis and angiogenesis, support for the blood-brain barrier (BBB), and prevention of the development of senile plaques in conjunction with cell replacement as shown in Figure 7 (De Gioia et al., 2020).

Spinal cord injury (SCI0) has been investigated for potential treatment via stem cells as it generally leads to loss of complete or partial motor function. Medical science has yet to come with an answer to treat SCIs. Neuroprotective therapy via Neuronal Stem Cell transplantation has been suggested as a prospective therapeutic approach towards SCIs. A recent investigation employing a murine model has imparted enhanced clarity regarding the role of neural stem cell (NSC) transplantation in the context of spinal cord injury (SCI) treatment. Xiang li et al. investigated whether wnt4 protein in conjunction with NSC transplantation can enhance motor function recovery in SCI mice. The results of this *in-vivo* experiment showed that the SCI mouse with wnt4 injections showed much more locomotory function after about 2 months’ time than the other placebo groups. It was concluded that NSC transplantation in conjunction with wnt4 protein undoubtedly induced neurogenesis and neuronal differentiation via activation of wnt pathway and inhibition of Notch pathway in NSC ultimately aiding to recovery of the SCI mice (De Gioia et al., 2020). As the mice-model has been long trusted for human drug and therapeutic developments for a long time due to similar physiological and pathological characteristics between mice and humans, this study gives a great platform for starting experiments on human subjects with SCIs (Rosenthal and Brown, 2007).

**FIGURE 8:**
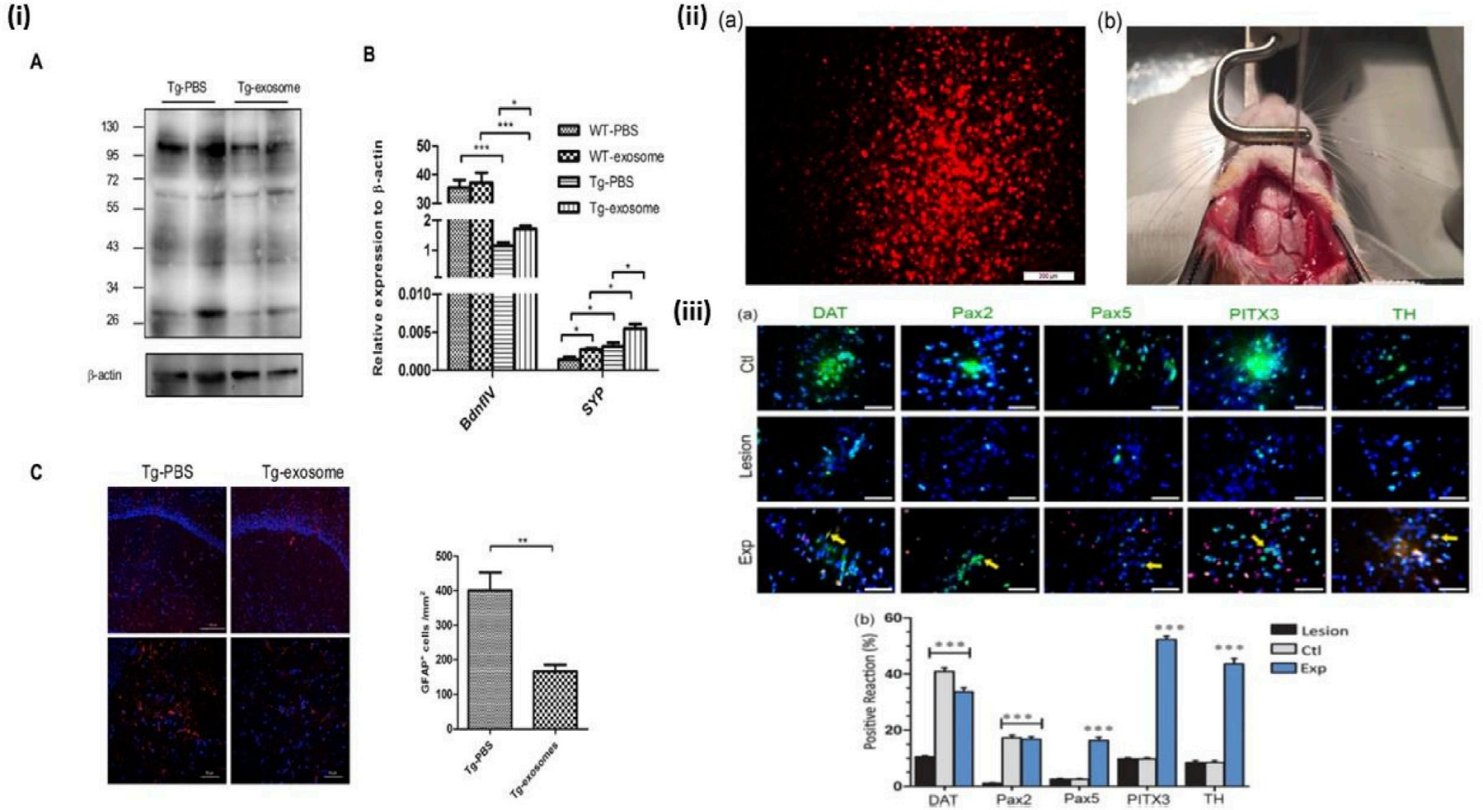
MSC-exosomes diminish Aβ plaque accumulation, suppress astrocyte activation, and enhance the expression of memory- and synapse-associated genes. **(i)** Transgenic or wild-type mice were administered MSC-exosomes or PBS for 4 weeks, after which homogenised brain tissues were subjected to analysis using SDS-PAGE and immunoblotting. A representative Western blot of soluble Aβ, immunoprobed with Aβ antibody and visualised using ECL, is presented. Internal control: β-actin. Quantitative RT-PCR findings of plasticity-associated genes from extracted hemispheres. Representative confocal micrographs of sagittal brain slices, immunolabeled with GFAP, within the hippocampus. Red indicates GFAP; Blue denotes DAPI. Scale bar: 100 μm (top), 50 μm (bottom). Quantification of GFAP + cells within a designated area of interest (Area = 0.05 mm^2^). Data are presented as mean ± SEM (p = 0.0043; **p < 0.01 according to Student’s t-test) (Chen et al., 2021). **(ii)** (a) The DiI-labeled OE-MSCs prior to transplantation (red cells), (b) The micrograph of rat calvaria for the transplantation of DiI-labeled stem cells using stereotaxic injection. DiI, 1,1‐dioctadecyl‐3,3,3′3′‐tetramethyl indocarbocyanine perchlorate; OE‐MSCs, olfactory ectomesenchymal stem cells (Simorgh et al., 2019). **(iii)** (a) Assessment of TH, DAT, PITX3, PaX2, and PaX5 expression using immunohistochemistry. The nuclei were labelled with DAPI (blue), whereas the cells exhibited green staining upon exposure to dopaminergic marker antibodies. The control group (Ctl) exhibited normal expression of dopaminergic markers in the right SNpc region. In contrast, the lesion group demonstrated a significant reduction in the expression of these markers compared to other groups within the same region. Conversely, the experimental group (Exp), which received injections of OE‐MSCs DiI+ (red cells), showed an increase in dopaminergic marker expression relative to the lesion group, with arrowheads indicating double-labeled cells. The proportion of positive responses to midbrain dopaminergic neurone markers (TH, DAT, Pax2, Pax5, and PITX3) was analysed across the groups (control, lesion, and experiment) in the right SNpc, republished with permission from Simorgh et al., 2019, Chen et al., 2021. Copyright 2019, Wiley.

Another neuronal disorder which has been investigated for potential MSC treatment is AD. 50%–70% of dementia cases are caused by the progressive neurodegenerative condition known as AD (Cummings et al., 2021). Within the framework of current hypotheses, the etiology of Alzheimer’s disease (AD) is posited to stem primarily from the accrual of neurologically detrimental amyloid beta-protein (Aβ) within the central nervous system (CNS). This hypothesis suggests that the cumulative presence of Aβ, characterized by its toxic properties, serves as a pivotal instigator of the pathophysiological processes underpinning AD (Ma et al., 2022). Chen YA et al. recently provided important insights on how AD can be treated in mice model via MSC-exosome mediated stem cell therapy highlighting details in Figure 8. Both *in vivo* and *in vitro* assays were performed which yielded the following results: Upon administration of MSC-exosomes it was found that there was considerable amount of Aβ Plaque degradation in the brain of neuronal network and restoration of cognitive function of the mice. FDG-PET imaging revealed a notable enhancement in brain glucose metabolism, which corresponded with a substantial improvement in cognitive functions. These findings were in concurrence with the observed upregulation of genes associated with memory and synaptic plasticity subsequent to the treatment (Chen et al., 2021). A similar study completed by Gong et al. demonstrated that Rat Adipose Tissue Derived-MSC when administered on the hippocampus of PSS/PS1 mice (Transgenic mice showing AD disease), induced neurogenesis in conjunction with alleviated cognitive development and neuroblast formation. Zheng et al. experiment further verified this study by administration of Amniotic-MSCs in the hippocampus of PSS/PS1 mice. Results were conclusive of the fact that there was definite decrease in Aβ peptide which is a hallmark of AD and neurogenesis was also observed (Zheng et al., 2017). In conclusion, stem cell-based neuroprotective therapies hold promising potential for addressing Alzheimer’s disease. These findings pave the way for future research and clinical exploration, urging the development of targeted interventions to mitigate neurodegeneration in AD.

Numerous animal studies have reported the neuroprotective properties of exosomes derived stem cells (SC-exos) and exosome-coated nanoparticles for treating NDs as the exosome possess unique characteristics such as biocompatibility and exhibit low immunogenicity. Exosome can traverse the BBB, making them suitable for delivering drugs and biomolecules to the CNS, with their surface markers enabling selective interactions. Neurotherapeutic strategies with SC-exos to treat AD, include anti-amyloid therapies. Mesenchymal stromal stem cell-derived exosomes (MSC-Exos) are currently under clinical investigation for regenerative and neuroprotective medicine (Jain et al., 2025).

Individuals diagnosed with Parkinson’s disease are classified as belonging to the second group of neurodegenerative disorders, following Alzheimer’s disease (Simorgh et al., 2019). In Parkinson’s disease (PD), the normal secretion of dopamine by striatum cells is impaired, leading to various abnormal movement symptoms such as rigidity and tremors. No cure of Parkinson’s disease has been reported yet. Studies on implementation of stem cells on PD has also surfaced. Principal cause of PD is accredited to loss of dopaminergic cells (Alizadeh et al., 2013). Simorgh et al. administered Olfactory Ectomesenchymal stem cells (OE-MSCs) in transgenic mice suffering from PD. After the labeling of OE‐MSCs with DiI, the MSCs were administered to the right SNpc with the help of a stereotaxic frame. The grafted cells were reported to survive for 2 months and showed expression of dopamine neuron markers. The expression of genes such as DAT, PAX5, PAX2 were much higher in the grafted group of mice as compared to the placebo group. The motor deficits in PD mice were reported to be corrected in this timeframe. This study gives a fascinating insight of potential therapeutic approaches against PD in human subjects (Simorgh et al., 2019).

Another therapeutic strategy with hNSC-Exos involves encapsulating dopamine or dopamine agonists in SC-Exos, ensuring targeted delivery to dopaminergic neurons. Exosome derived from Bone-marrow MSCs (BM-MSCs) consisting of Gli1 protein were found to inhibit Sp1 signaling, thus decreasing microglial activation and neuronal apoptosis. This finding suggests a possible mechanism for mitigating cellular inflammation and death in PD. Exosome derived from MSCs have been found to modulate cholesterol metabolism in neuronal cells via the Wnt5a - LRP1 axis, thus ameliorating cognitive function in a progressive PD model. It has been also found that human umbilical cord MSC-derived Exosome loaded with BNDF enhance neuroregeneration and functional recovery in the PD model as well (Wang Z. et al., 2023).

Stem cell transplantation in mice models has also had positive results in other neurodegenerative disorders such as Huntington’s disease. After 10 weeks of intrastriatal Quinolinic Acid (QA) injection, Lin et al. demonstrated that mice that had human BMSC transplantation had a significant reduction in motor impairment and an enhanced survival rate. In the QA-lesioned striatum, transplanted BMSCs were able to proliferate and induce neuronal differentiation. But motor function improvement was not observed (Lin et al., 2011).

### 4.3 Anti-neuroinflammatory activity

Neuroinflammation is an important accelerator of neurological disorders (Bersano et al., 2023). It involves the immune system dysregulation within the nervous system. M1 and M2, two different microglial cell phenotypes, play crucial roles in this process. Pro-inflammatory M1 phenotypes release cytokines that increase neuroinflammation such as IL-6, TNF-a. M2 phenotype, in contrast, takes on anti-inflammatory roles that assist trophic support, tissue healing and angiogenesis. Neurodegenerative disorders are influenced by dysregulation of the M1/M2 balance (Saqib et al., 2018). Hence, modulating microglia towards the M2 phenotype while suppressing the M1 phenotype emerges as a promising therapeutic avenue in addressing neuroinflammatory conditions (Liu et al., 2019). A few studies have shown that MSC treatment can indeed improve neuroinflammation by switching the polarization of microglial cells towards M2 phenotype (Teixeira et al., 2017). It was found that LPS-neuroinflammatory mice when treated with MSCs showed significant reduction in pro-inflammatory gene (TNF-a, IL-6 etc.) expression and improvement in expression of anti-inflammatory genes such as IL-10 was observed. Further analysis suggested an improved expression of M2 markers such CD206 in the group of mice which received MSCs in comparison with the placebo mice (Liu et al., 2019). This study further backs point put forward by the studies done by Teixeira et al. and establishes MSC treatment as a promising prospect of therapeutic approach in neuroinflammatory disorders.

**FIGURE 9:**
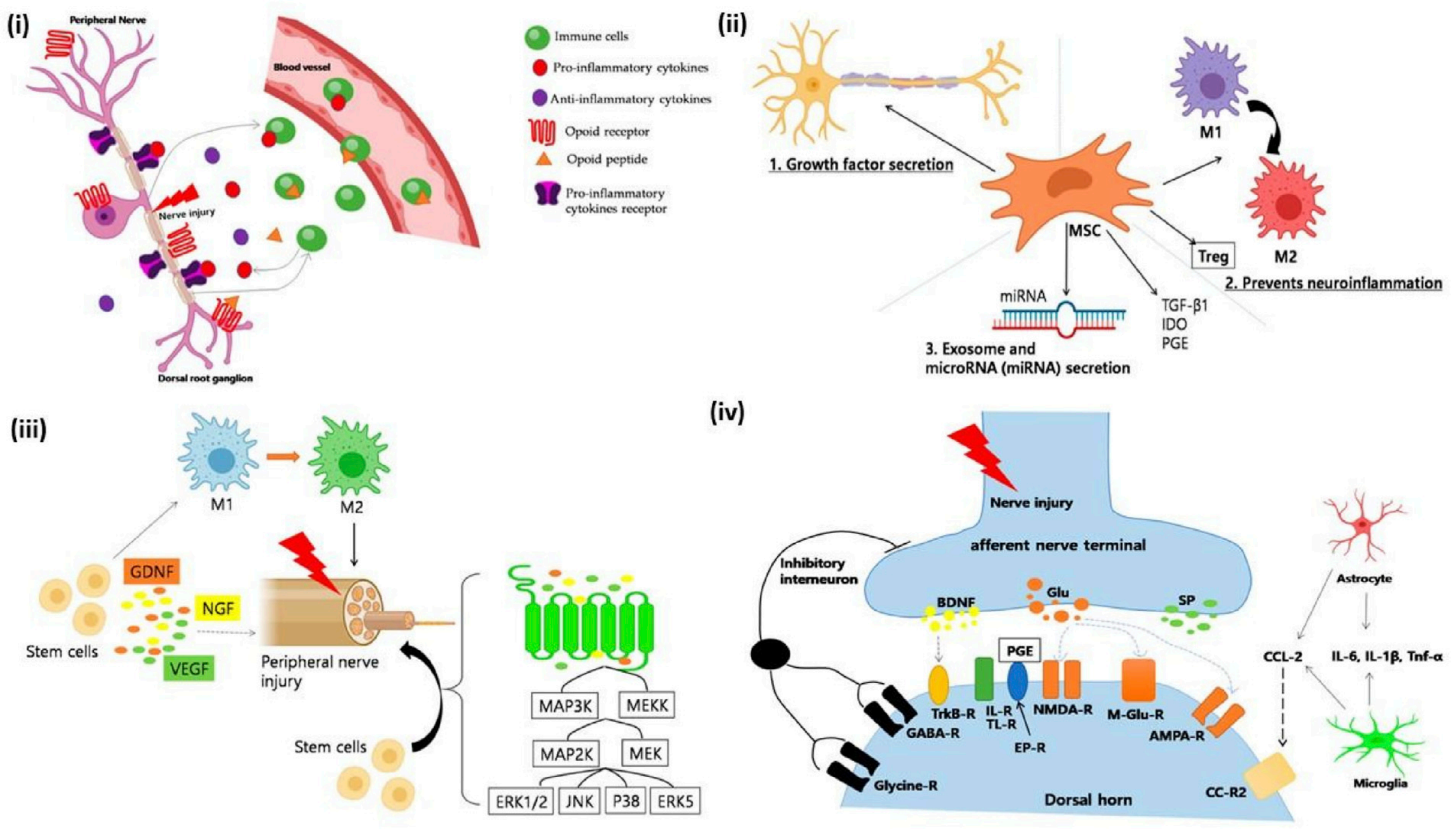
**(i)** Immune cells modulate neuropathic pain. After a peripheral nerve injury, immune cells release proinflammatory cytokines (e.g., TNF-α, IL-1β) that interact with receptors and cause pain. Opioid peptides, which help to relieve pain, can also be produced by immune cells. Peripheral opioid receptors are found in the dorsal root ganglia and go to the nerve injury site. Once there, opioid peptides activate receptors and reduce neuropathic pain. **(ii)** A schematic depicting the mechanism of neuropathic pain healing facilitated by mesenchymal stem cells (MSCs). Several distinct mechanisms are involved: (1) Growth factor secretion; MSCs produce neurotrophic growth factors such as GDNF, VEGF, and BDNF. Neurotrophic growth factors have been shown to promote neuronal survival in cases of neuropathic pain. (2) Reduced neuroinflammation; MSCs significantly influence the immune system and help in wound repair. Interestingly, MSCs can be anti-inflammatory or proinflammatory depending on the environment in which they dwell. In an inflammatory environment, MSCs produce TGF-β1, IDO, and PEG, converting macrophages/microglia from proinflammatory to anti-inflammatory M2 phenotypes. Furthermore, MSCs can stimulate the upregulation of T cells, which are known to play an important role in pain modulation (3), as well as the production of exosomes and microRNAs. MSCs produce biological substances via extracellular vesicles (EVs), which include microvesicles and exosomes. EVs include thousands of proteins, messenger RNA, and/or microRNA, which have been shown to promote neuronal development. **(iii)** A schematic picture of how stem cells work in peripheral neuropathic pain. (a) Anti-Inflammatory Regulation: Stem cells drive macrophage polarization to anti-inflammatory phenotypes. M2 macrophages increased following MSC therapy, but genes associated with M1 macrophages decreased. (b) Neuroprotection and axonal myelin regeneration. Stem cells also have anti-inflammatory properties via the mitogen-activated protein kinase (MAPK) pathway. After nerve injury, signals from damaged axons activate the extracellular signal-related MAPK signal pathway in Schwann cells. MSCs inhibited the expression of pERK1/2 in CCI-induced dorsal root ganglion (DRG) cells. Furthermore, VEGF, GDNF, and NGF are essential nerve regeneration regulators that can help and stimulate the formation of newly formed nerve fibres. **(iv)** Mechanisms linked with nerve damage at the synapse of peripheral nerves and spinal cord dorsal horn neurones. (a) Weakened and reversed central sensitisation. Following nerve damage, the release of excitatory amino acids (glutamate) in the spinal dorsal horn significantly increased, and the excitatory N-methyl-d-aspartate (NMDA) receptor (NMDAR) is continuously activated. According to reports, bone marrow stromal cells (BMSCs) can decrease the production of NMDA receptors and protect them from glutamate excitotoxicity, reducing mechanical hyperalgesia. (b) The inhibition of glial cell activation. Stem cells can effectively prevent the activation of glial cells like microglia and astroglia. They also prevent MAPK signal pathway activation in activated glial cells. (c) Reduced apoptosis and autophagy in spinal cord cells. The stimulation of intermediate inhibitory neurons causes the release of GABA, a neurotransmitter that inhibits postsynaptic neurons via membrane hyperpolarization, reproduced with permission from ref. (Joshi et al., 2021), copyright 2021 MDPI.

Neuroinflammation often leads to a complex condition known as Neuropathic Pain (NP). NP treatment has proven to be a challenging task since the concept of its underlying mechanism is vastly unclear and vague. Inflammatory immune cells play a crucial role in NP. Especially neutrophils which are one of the immune systems most potent killers, has been reported to show major influence in NP development in mice model (Gong et al., 2016). Macrophages as mentioned earlier have also been reported to release pro-inflammatory cytokines (Saqib et al., 2018). In study conducted in Chronic Constriction Injury sciatic nerve injury model, when AD-MSCs were administered at the point of inflammation, it was found that there was a significant suppression of IL-6 and IL-1β. Subsequently IL-10 levels improved suggesting potential repression of the inflammatory pathway and initiation of anti-inflammatory pathway (Joshi et al., 2021). These results coincide with the results of Liu et al. and further strengthens the point that MSCs have invariant neuroinflammatory suppression mechanism. Figure 9 summarizes key mechanisms of macrophage polarization, glial inhibition, trophic factor secretion, and suppression of excitotoxic signaling in stem cell therapy that improves neuropathic pain, promoting neuronal repair both peripherally and centrally by modulating immune responses, reducing inflammation.

Multiple Sclerosis (MS) stands as a prominent neuro-immunological complication, characterized by the disruption of effective communication between the central nervous system (CNS) and the peripheral body, attributable to an autoimmune assault on the myelin sheath enveloping nerve fibers. This autoimmune phenomenon precipitates the degradation of myelin, a protective insulating layer indispensable for the swift and efficient propagation of nerve impulses. The consequential deterioration in signal transmission along neural pathways culminates in an array of debilitating neurological manifestations. In the etiology of MS, intricate immunological dysregulations intertwine with neurological pathophysiology, thereby underscoring the complexity of this disorder. There is presently no cure for MS, and its precise cause is still not entirely understood (Trapp and Nave, 2008). Several clinical trials have been reported on the use of stem cell therapy in MS patients and positive results have been retrieved through many of those studies. The initial small clinical trials in multiple sclerosis have already demonstrated the safety of MSC use, opening the door to phase II investigations that examine the biological impact of MSCs on disease activity indicators. A Phase I open-label clinical trial employing intrathecal (IT) administration has yielded preliminary findings of significant interest. Within this trial, a cohort of 20 patients diagnosed with multiple sclerosis (MS) and exhibiting varying degrees of disability were selected to participate. The intervention involved the intrathecal infusion of autologous mesenchymal stem cell-neural progenitors (MSC-NPs), with three distinct dosages administered at 3-month intervals. Notably, each dosage encompassed a population of up to 10 million cells. In the nascent stages of this trial, an initial safety assessment was conducted, focusing on the first five enrolled participants. Encouragingly, these initial findings underscore the safety profile and tolerability of the administered therapy within this subgroup. This noteworthy outcome holds implications for the advancement of therapeutic strategies in the realm of MS treatment. Further investigation in subsequent phases holds the promise of elucidating the broader therapeutic potential of MSC-NP-based interventions in mitigating the impact of MS-associated disabilities (Harris et al., 2018). In the realm of multiple sclerosis (MS) research utilizing animal models, mesenchymal stem cells have demonstrated a profound influence on both the innate and adaptive components of the immune system. A notable advancement is exemplified in a phase II clinical trial focusing on secondary progressive MS, wherein comprehensive assessment encompassed visual and neurophysiological efficacy parameters. Additionally, a randomized controlled trial, albeit conducted on a limited cohort of relapsing-remitting MS patients unresponsive to a minimum of 1 year of established treatments, holds significance. It is pertinent to acknowledge that a predominant proportion of documented trials assume the form of uncontrolled open-label phase I investigations. This extensive array encompasses secondary progressive MS, progressive MS, relapsing-remitting and secondary progressive MS, as well as patients presenting with active yet non-specific clinical manifestations (Connick et al., 2011).

Though several conventional therapeutic attempts for HD based on pathogenic mechanisms involving gene silencing, transglutaminase inhibition or upregulation of autophagy have been researched on, unfortunately still fail to meet the criteria for clinical translation (Consortium, 2021). MSC transplantation have gained substantial attention as potential therapeutic strategy for HD, with its repair mechanisms attributing to anti-neuroinflammatory, immunoregulatory, neutrophic and antiapoptotic pathways (Liang et al., 2023). Bone Marrow -derived MSCs were intrastriatally transplanted in three different HD rodent models and it has been demonstrated that BM-MSCs could reduce striatal atrophy, cell loss by chemokine secretion to promote endogenous progenitor cell recruitment and improvement in neuronal cell differentiation (Connor, 2017). Cell replacement in SC treatment for this autosomal genetic disorder have also the ability to affect multiple pathogenic pathways through paracrine release of neuroprotective and immune modulatory factors. With the discovery of iPSC reprogramming technologies, transplanted HD iPSC-derived NSCs were generated and investigated in QA lesion model, resulting in neuronal differentiation expressing markers of MSNs and improved functional outcome (Liang et al., 2023). At present, MSCs appear to exert positive findings in HD SC therapy through their ability to enhance compensatory neurogenesis, modify immune cell dysfunction, decrease neuroinflammatory and apoptosis, hence herald a promising new era for HD SC research strategies (Connor, 2017).

## 5 Nanomaterials in stem-cell generation and scaffolding

The goal of regenerative medicine is to restore the functionality of tissue or cells that have been damaged by aging, infection, or injury. Stem cells have the capability to be exploited for tissue restoration and healing due to their capacity for self-renewal and cell-type differentiation. The past few decades have seen significant advancements in our knowledge of the nature of stem cells as well as our capacity to control their growth and division to produce healthy tissues. In the field of regenerative medicine, the combination of stem cells and scaffolding techniques holds great promise for addressing a wide range of health conditions (Liliang et al., 2017). Scaffolds are essential for promoting the expansion, multiplication, and transformation of stem cells. They produce a three-dimensional (3D) environment that closely mimics the structure of actual tissues by imitating the extracellular matrix (ECM). These scaffolds can be classified into three categories: natural, synthetic, and hybrid, each of which offers distinctive characteristics (Zhao et al., 2013).

**FIGURE 10:**
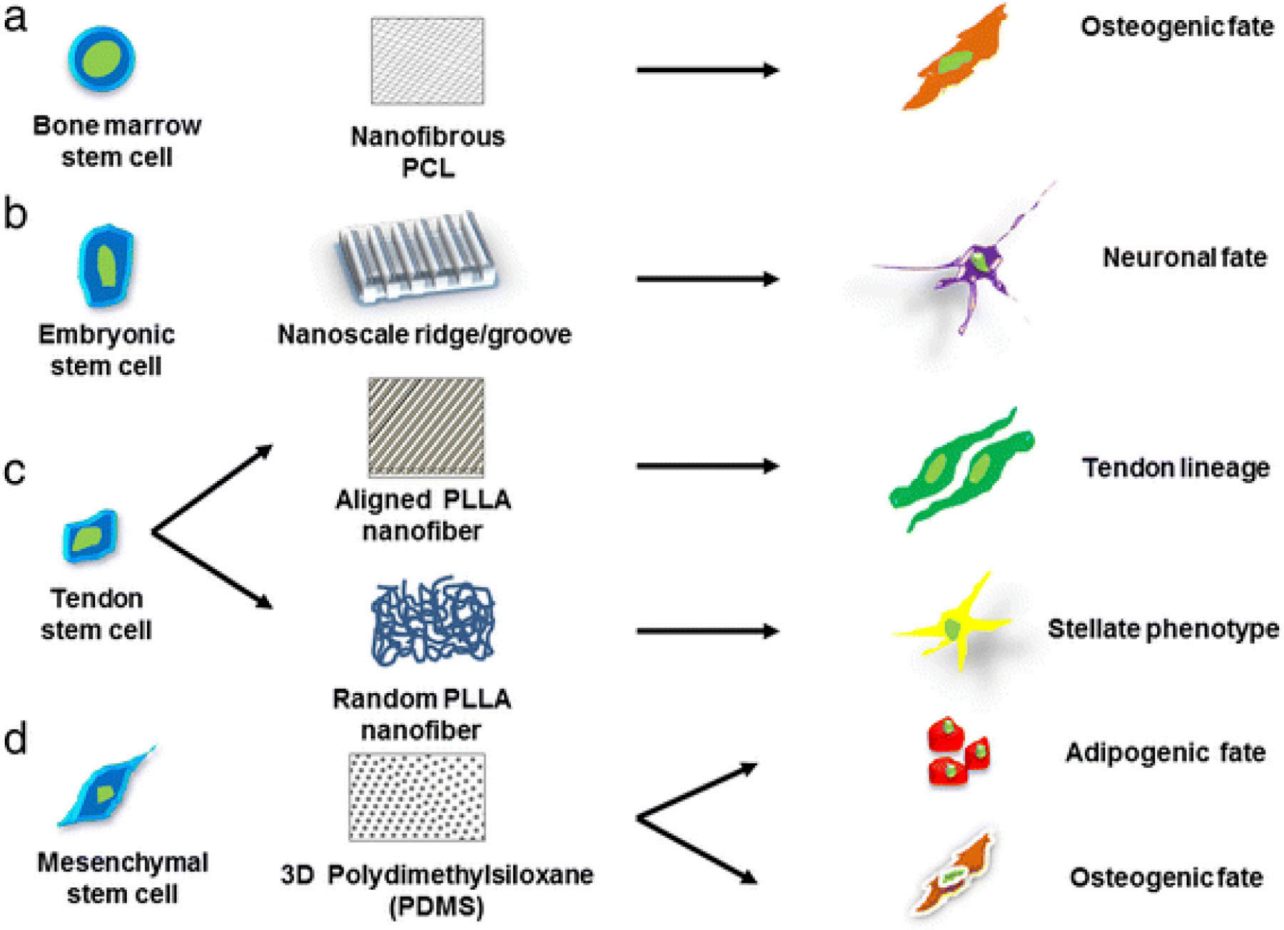
Schematic illustration defining the significance of different scaffold architectures in identifying the specific lineage of stem cells. Stem cells cultured on different nanostructured scaffolds produce distinct differentiated cell types, such as **(a)** bone marrow stem cells grown on nanofibrous PCL scaffold promotes osteogenic fate, **(b)** embryonic stem cell cultured on nanoscale ridge or groove promote neural fate, **(c)** tendon stem cells cultured on aligned and random PLLA scaffolds guide tendon and stellate lineage, respectively, **(d)** mesenchymal stem cells on PDMS promote osteogenic and adipogenic fate, reproduced with permission from ref (Krishna et al., 2016). copyright 2021 MDPI.

Natural scaffolds are very similar to the native ECM since they are made of biological materials including collagen, fibrin, chitosan, and hyaluronic acid. They are naturally bioactive and biocompatible, which encourages cell attachment and signaling. Natural scaffolds can be modified using nanomaterials to improve their bioactivity, mechanical characteristics, and controlled release of bioactive compounds. In contrast, polymers like poly (lactic-co-glycolic acid) (PLGA), polyethylene glycol (PEG), and polycaprolactone (PCL) are used to create synthetic scaffolds. These scaffolds have adjustable surface functions, mechanical properties, and durability. Synthetic scaffolds give scientists complete control over the scaffold’s properties, enabling customization to fit particular needs in regenerative medicine. Hybrid scaffolds were created to incorporate the benefits of both natural and synthetic scaffolds. It uses natural polymers to improve their bioactivity and synthetic polymers for mechanical stability. In addition to providing an adaptable structure for stem cell growth and differentiation, this combination also promotes regulated tissue breakdown (Krishna et al., 2016). The type of scaffold used will dictate the stem-cells growth as Figure 10 describes the effect of matrix geometrical force on cell fate helping in tissue-specific regeneration. This is because stem cell viability is strongly controlled by its microenvironment, which include both internal and external cell communication. Biophysical signals, in addition to chemicals and growth factors, are a significant part of the external stimuli that control stem cell traits. Chemical signals are provided by the components used to make scaffolds, and biophysical cues are provided by the scaffold’s structure (Dubey et al., 2022). Hence, it is important to make scaffolds in the right way, and this process can be improved by using nanomaterials.

Nanomaterials have emerged as powerful tools in this domain, revolutionizing the generation and manipulation of stem cells while providing an optimal environment for their growth and development. The various nanomaterials used for this purpose are nanofibers, nanoparticles, carbon-based nanomaterials, nanogels, and nanowires. For e.g., bioactive chemicals or growth factors can be encapsulated in nanoparticles like liposomes or polymeric nanoparticles and delivered to stem cells within scaffolds in a targeted manner. The proliferation, differentiation, and tissue regeneration of stem cells are improved by this regulated release (Kerativitayanan et al., 2015). Monteiro et al., demonstrated this by their work on pDNA loaded liposomes. In this study, the aim was to develop a system for delivering genes that combined electrospun nanofiber mesh (NFM) as a framework for building tissues, with liposomes acting as vectors bound to the scaffold. The rate of metabolism and translation of human bone marrow-derived mesenchymal stem cells (hBMSCs) grown on these bound liposomes were significantly increased. It caused cultured hBMSCs to produce genes for an extended period of time. Additionally, the increased expression of additional bone-forming markers in media devoid of bone-forming supplements allowed hBMSCs to differentiate into osteoblasts. These results show that nanomaterial on scaffold can result in long-term transcription *in vitro* and improve the activity of scaffolds for tissue remodeling (Monteiro et al., 2014).

By nano structuring scaffold surfaces, nano topography is created, providing fine-grained control over stem cell behaviour. Nanostructured surfaces have the ability to direct stem cell proliferation, differentiation, and alignment, promoting the growth and organisations of functional tissues. The nanoscale characteristics of the scaffold can be altered to affect cellular responses and direct the formation of new tissues. This alteration is primarily done during the making of the nanomaterial. Specialized tools and methods are needed for the precise manufacturing of nanoscale scaffolds. Using a high-voltage electric field, the widely used technique of electrospinning draws polymer solutions into ultrafine fibres, which are subsequently gathered to form a scaffold. The creation of nanofiber scaffolds using electrospinning methods is a very important role played by nanomaterials. Electrospun nanofiber scaffolds offer the best conditions for stem cell adhesion, migration, and differentiation because they closely mirror the ECM’s fibrous structure. Furthermore, the incorporation of carbon nanotubes to these scaffolds improves their electrical conductivity and mechanical robustness. This opens up opportunities for building functional brain tissues by enabling electrical stimulation for neural stem cell development (Dubey et al., 2022). This method makes it possible to create scaffolds with tightly controlled fibre sizes and high surface-to-volume ratios, which improve cellular interactions within the scaffold. A different cutting-edge method called 3D bioprinting uses a combination of cells, biomaterials, and growth factors to produce intricate 3D structures. Bioinks are layer-by-layer deposited using specialized printers, providing fine control over scaffold architecture and cell positioning. The construction of complex brain tissue constructs using 3D bioprinting holds considerable promise for easing the incorporation of neural cell lines into engineered systems (Krishna et al., 2016).

The discovery of neural stem cells (NSCs), which disproved the theory that the adult nervous system loses the ability to regenerate new neurons, was one of the ground-breaking discoveries in neurobiology over the past 2 decades. These multipotent stem cells have the ability to differentiate into a range of neural cells, such as neurons, astrocytes, and oligodendrocytes, provide the flexibility needed to heal and restore the neural network. As a result, they have been anticipated as an exciting therapeutic approach for a variety of brain diseases in order to promote functional recovery. Physical characteristics have a crucial influence in the efficiency of brain cell lines. Electrical receptivity in neural cells enables them to send out and analyze electrical impulses. For the integration of brain circuits within tissue-engineered constructions, this property is essential. Successful brain tissue regeneration depends on synaptic connectivity, the development of complex connections between neurons that allows communication inside the nervous system (Dubey et al., 2022). For example., a study conducted by Vafaei et al., uses PCLF electrospun random nanofiber scaffolds for the neural stem cells extracted from mice brain. It showed that these scaffolds significantly increased the growth and proliferation of cells into the neural phenotype as compared to control (Vafaei et al., 2021). Another study suggests that disseminating the stem cells via scaffolds produced from precise and approved collagen material may improve the effectiveness and growth of emerging neuronal stem cell-based scaffold treatments (Kourgiantaki et al., 2020).

Hence, nanomaterials play a crucial role in stem cell generation and scaffolding, facilitating the development of regenerative medicine strategies. By harnessing the unique properties of nanomaterials, researchers can create tailored scaffolds for neural cell lines, enabling the growth, differentiation, and organization of functional neural tissues. Advancements in scaffold fabrication techniques will continue to drive innovation and bring us closer to realizing the full potential of stem cell-based therapies.

## 6 Nanomaterial conjugated stem-cell therapy

Stem cell therapy offers immense potential for treating neurodegenerative illnesses, by helping to restore lost or injured neurons and encourage brain regeneration. There are key advantages of using stem cell therapy especially in Neurodegenerative disorders. For example, in cases where the specific neuronal populations gradually disappear, it may be possible to replace these dying neurons with new, functional ones by stem cells, which will promote brain healing and cause the patient to regain cognitive and motor abilities. Stem cells also possess immunomodulatory characteristics that allow them to control the immune response in the brain. This regulation can prevent additional harm to neurons by reducing neuroinflammation, a defining feature of neurodegenerative disorders. Additionally, numerous neurotrophic substances are secreted by transplanted stem cells, supporting the survival and functionality of preexisting neurons. These elements support a healthy milieu for neuronal function, perhaps delaying or decreasing the progression of illness (Wang et al., 2009).

The effectiveness of stem cell therapy, however, depends on overcoming a number of obstacles, including effective stem cell transport, survival, and integration into the diseased tissue. Nanomaterials are essential for maximizing the benefits of stem cell therapy because they have special features that help with stem cell distribution, protection, and monitoring. Firstly, nanomaterials act as transporters, enabling efficient and focused transportation of stem cells towards the brain’s injured areas. The homing of stem cells to the appropriate region is facilitated by the functionalization of nanomaterials with ligands unique to the diseased tissue. Secondly, they can be designed to release therapeutic chemicals in a regulated manner, like growth factors, which encourage stem cell differentiation and tissue regeneration at the target site. Lastly, transplanted stem cells are shielded by nanomaterials both during transport and after transplantation, increasing their longevity and lowering cell death in the challenging microenvironment of the brain (Vissers et al., 2019).

A study conducted by Torres-Ortega et al. which targeted Parkinson’s syndrome, was involved in developing and characterizing a composite hydrogel that has been modified with nanoparticles containing mesenchymal stem cells and neurotrophic factor produced from glial cell lines. The nanoparticle had a dual role by acting as a perfect carrier and also as a helper in crosslinking of the hydrogel, whereas the hydrogel helped in protection and sustained release of the materials. The composite hydrogel is a promising choice for drug and cell delivery to the brain due to its adequate endurance, superior regeneration abilities, strong biocompatibility, and capacity to enhance mesenchymal stem cells regeneration capability (Torres-Ortega et al., 2022). Another study conducted by Koudehi et al. created a unique nano bioglass/gelatin conduit (BGGC) for the healing of peripheral nerves. The bioglass nanoparticles were made through the sol-gel method, and they were then characterized. The findings demonstrated that BGGC is a potential option for peripheral nerve repair since it had excellent durability and a strong ability to heal nerves in a rat model (Koudehi et al., 2014).

Recent advancements in regenerative medicine have highlighted the nanotechnology -enhanced stem cell therapy showing promising multifunctional approach for AD, aiming to overcome the limitations of conventional stem -cell treatments. The elevated presence of Aβ oligomers in the diseased brain milieu has been shown to suppress the neuronal differentiation potential of transplanted stem cells, further restricting their regenerative impact. Thus. Integration of stem cells with engineered nanomaterials, this strategy not only supports neural regeneration but also enhances the clearance of beta-amyloid (Aβ) plaques - one of the key pathological hallmarks of the disease. Genetically engineered NSCs capable of stably and continuously expressing neprilysin (NEP) are developed to enhance (Aβ) degradation and NSC survival in the brain. A nano formulation composed of PBAE-PLGA-Ag_2_S-RA-siSOX9 (PPAR-siSOX9) has been developed to effectively deliver both genetic and pharmacological agents, aiming to counteract the harmful conditions of the Alzheimer’s disease microenvironment and to promote neuronal differentiation of the NEP-expressing NSCs (Sivandzade and Cucullo, 2021a).

Nanomaterials-conjugated stem cell therapy shows significant promise for revolutionizing regenerative medicine and enhancing the lives of patients with neurological diseases especially as and when research into nanomaterials and stem cell technology advances. This innovative approach harnesses the power of nanomaterials as carriers, guiding stem cells to specific sites within the central nervous system for neurorecovery, and helping in their modulation and monitoring.

### 6.1 Stem cell delivery for neuronal recovery

Due to the restricted ability of neurons to regenerate, recovering damaged or diseased neurons in the central nervous system (CNS) is a difficult task. A possible method to restore brain function and encourage neuroregeneration is stem cell treatment. However, effective and accurate stem cell transport to the afflicted brain areas is essential for the successful implementation of stem cell therapy. Delivering stem cells to the site of damage or degeneration has been made much more efficient and focused in recent years thanks to the incorporation of nanomaterials. The capacity to precisely target particular CNS regions is one of the key components of stem cell administration for neuronal rehabilitation. Nanomaterials can be modified with ligands or coatings that recognize particular receptors or markers on injured neurons or neural support cells in order to deliver stem cells. This increases the likelihood of successful engraftment and integration by enabling the exact homing of stem cells to the site of injury (Bonosi et al., 2022).

Treatment of spinal cord injuries is one example of how nanomaterials-conjugated stem cell transport for neuronal regeneration is used. In a pioneering study, researchers designed magnetic nanoparticles as carriers to transport neural stem cells to the injured spinal cord. It was successful to load stem cells with designed SPIONs that ensure adequate attractive magnetic fields. Additionally, the magnetic technology made it possible to direct the SPION-labelled cells quickly and precisely to the site of the injury. A good connection was found between the calculated distribution of the magnetic forces applied to the transplanted cells and the histological study of cell dispersion throughout the cerebrospinal channel. The outcomes imply that the suggested non-invasive magnetic technology is capable of focused targeting and quick distribution of stem cells (Boukhatem et al., 2020). Nanocarriers such as polymer-lipid hybrid nanoparticles (PLNs) delivering retinoic acid (RA) and siRNA to enhance NSCs differentiation and target AD pathology, provides biodegradability and sustained release. The co-delivery of RA and siRNA to the brain modulates the stem cell microenvironment and boosts endogenous regeneration. (Zhou and Kihara, 2023).

The substantial number of metastases, accessibility, and transplanted stem cell death are this approach’s most significant drawbacks. Therefore, a likely remedy to this issue is to induce indigenous neural stem cells (NSC) *in situ* in order to encourage their development into neural cells (Masoudi et al., 2020). Researchers have fully utilized 2D nanomaterials in the area of neuronal recovery, which have demonstrated benefits in the identification and management of disorders connected to nerve injury. This has encouraged their widespread utilization in the domain of nerve growth and healing (He et al., 2022). Bioactive 2D nanomaterials for neural repair and regeneration. Nanomaterials serve as transporters, but they also help transplanted stem cells stay safe and thrive in the unfriendly milieu of the wounded CNS. Nanomaterials can protect stem cells from immune reactions and lower the chance of rejection, increasing the transplanted cells’ long-term survival and therapeutic potential. Additionally, nanomaterials can be designed to deliver medicinal products or growth factors in a regulated manner, promoting the differentiation of stem cells and tissue regeneration. With the help of this controlled release, stem cells can differentiate into the desired neuronal cell types, such as dopaminergic neurons in Parkinson’s illness or cholinergic neurons in Alzheimer’s disease (Ochalek et al., 2016). Retinoic acid (RA), one of the compounds that can transform stem-cell offspring into desirable lineage-specific progenitors, is an excellent option to trigger gene transcription that is involved in cell growth, development, and apoptosis. Endogenous stem cells can be forced out of their neurogenic niche, which increases the brain’s capacity for regeneration (Ferreira et al., 2008).

With several promising methods now in clinical trials, the potential of nanomaterials-conjugated stem cell transport for neuronal rehabilitation goes beyond preclinical investigations. Nanomaterials provide special benefits in targeted distribution, stem cell protection following transplantation, and the development of a favorable milieu for tissue regeneration. Numerous studies show the enormous potential of stem cell therapy using nanomaterials to promote neuroregeneration and give patients suffering from CNS injuries and neurodegenerative illnesses fresh hope. Nanomaterials hold the key to unlocking the full therapeutic potential of stem cells and revolutionizing the practice of regenerative medicine in the field of neuroscience, according to ongoing research. However, it is also important to mitigate their limitations and focus on other effects of nanomaterials before actively employing this technique.

### 6.2 Neuronal-stem cell modulation

In the area of regenerative medicine, modulating neuronal-stem cell interactions has enormous potential, especially when combined with the use of nanomaterials in stem cell therapy. New options for treating neurological illnesses and encouraging brain healing can be unlocked by comprehending and utilizing the complex interaction between neurons and stem cells. The dynamic interaction between neurons and stem cells, which affects one another’s behaviour and functionality, is known as neuronal-stem cell modulation. The reason behind neuromodulation of stem cells is because the transplanted cells must rebuild exact synaptic communication, reorganize the extension style, and restore the deteriorated host neurons for stem cell therapy to be effective. However, implanted neurons can fail to accurately merge into the host brain circuits and effectively replace missing neurons, leading to unanticipated results and sometimes serious adverse effects. Additionally, transplanted neurons usually have aberrant firing patterns, which disrupts their functionality. Hence, the creation of additional methods to enhance stem cell growth and proliferation is crucial to facilitating a full recovery (Yuan et al., 2021).

The neurons and stem cells have direct physical interactions that play a key role. Neuronal processes, such as axons and dendrites, can come into direct contact with stem cells, facilitating the exchange of molecular cues and promoting cellular responses. Chemical interactions can also occur where neurons release a variety of bioactive molecules, including growth factors, cytokines, and chemokines, which can act on nearby stem cells (Baptista et al., 2014). There can also be extracellular matrix interactions that provide a complex microenvironment for neuronal-stem cell interactions. ECM components, such as laminin and fibronectin, can influence stem cell differentiation, neurite outgrowth, and synaptogenesis (Gattazzo et al., 2014). All these interactions module the growth and proliferation of stem cells harnessing the crosstalk between neurons and stem cells, researchers are exploring novel approaches like use of nanomaterials to tackle neurological disorders and promote neural tissue regeneration.

For neurodegenerative conditions such as Parkinson’s disease, Alzheimer’s disease, and amyotrophic lateral sclerosis (ALS), neuronal-stem cell regulation offers prospective therapeutic options. Strategies include boosting neuronal survival and functional recovery by paracrine signaling and cell replacement therapies, as well as increasing endogenous stem cell activation and migration to regions of brain injury. For the treatment of these diseases, nanomaterials are engineered to improve stem cell survival, differentiation, and integration causing treatment options to be optimized for the particular disease (Masoudi et al., 2020; Wei et al., 2023). For example, a study demonstrated that carbon-based nanomaterial had significant positive effects on neural stem cell cells (Ren et al., 2022).

Neuronal-stem cell modulation also shows promise in neural injury cases, such as spinal cord damage and traumatic brain injury, for accelerating tissue repair and functional recovery. Transplanted stem cells can interact with damaged brain tissue by trophic support, angiogenesis promotion, and immune response modulation. Additionally, endogenous stem cell activation and behavioral modification can support the regeneration of brain tissue. Nanomaterial-functionalized transplanted stem cells can interact with damaged brain tissue by trophic support, angiogenesis promotion, and immune response modulation. Real-time monitoring of the distribution and engraftment of transplanted cells is made possible by the non-invasive imaging and tracking of transplanted cells caused by the incorporation of nanomaterials into stem cell treatment (Tang et al., 2024). A study done by Debnath et al., showed that nanomaterials can operate as multifunctional drugs to reduce oxidative stress and even inhibit the aggregation of harmful proteins (Debnath et al., 2017). Xu et al., in their review article has also highlighted the use of nanomaterials for imaging and tracking of components in Neurodegenerative disorders (Xu et al., 2021). Learning and memory are greatly aided by neuroplasticity, the brain’s capacity to reconfigure and change in response to environmental stimuli. By encouraging the integration of stem cell-derived neurons into already-existing brain circuits, nanomaterial-conjugated stem cell therapy has the potential to improve neuroplasticity. Neurogenic niches, like the hippocampus, contain stem cells that are constantly producing new neurons. Modulation is crucial because it affects learning, memory, and cognitive function as a result of the interplay between these developing neurons and pre-existing neural circuits. Nanomaterial-conjugated stem cells can alter synaptic connections, encourage the creation of functioning brain networks, and aid in cognitive recovery by modulating the interactions between neurons and stem cells (Wei et al., 2023).

It is also worth mentioning that modern gene editing techniques like CRISPR-Cas9 provide precise tools for modifying relationships between neural stem cells. The control of paracrine signaling, neuronal development, and stem cell survival can all be improved through genetic manipulation. To alter signaling pathways and encourage desired physiological responses, molecular strategies like small molecules and microRNAs can also be used. These tools could be extensively used in the future for neuronal stem cell modulation (Damasceno et al., 2020).

### 6.3 Monitoring stem-cell therapy

We have seen that stem cell therapy has tremendous promise and offers fresh methods for treating a variety of illnesses and Neurodegenerative disorders. The effectiveness of stem cell therapies, however, depends on efficient monitoring to guarantee safety, improve treatment plans, and evaluate therapeutic efficacy. Stem cell therapy monitoring is the systematic assessment of a number of important factors, such as cell viability, engraftment, differentiation, integration, and long-term safety. Researchers and doctors can obtain vital data to guide treatment procedures and customize interventions for specific patients by using a multifaceted approach to monitoring (Hachimi-Idrissi, 2023). In the context of nanomaterial-conjugated stem cell therapy, monitoring becomes even more crucial to ensure the success of the therapy and to exploit the benefits of nanomaterials in enhancing stem cell behavior and therapeutic efficacy (Xu et al., 2011).

Drugs now available for various Neurodegenerative disorders cannot efficiently cross the blood-brain barrier (BBB), which results in a poor prognosis and ineffective therapy. Thus, the creation of innovative diagnostic techniques and treatment plans is urgently needed. Nanomedicine has received a lot of interest lately for the detection and treatment of illnesses of the central nervous system (CNS). Nanoparticles enable therapeutic components to pass the blood-brain barrier and combine delivery, visualization, and treatment in a single system, giving individuals a fresh chance (Luo et al., 2020). Key determinants of therapy success are stem cell’s viability and survival. Nanomaterials can be used in nanomaterial-conjugated stem cell treatment to increase stem cell viability and safeguard them during transplantation. Real-time visualization and tracking of transplanted stem cells are made possible by monitoring techniques such molecular probes and non-invasive imaging utilizing contrast agents based on nanomaterials. This offers important details about their survival, growth, and fusion with the target tissue. Notably, gold nanoparticles (AuNPs) and superparamagnetic iron oxide nanoparticles (SPIONs) can both be used as colorants in clinical live imaging because of their superior magnetic and visual characteristics (Egawa et al., 2015).

For efficient tissue regeneration, nanomaterials can control stem cell behavior and direct their development into particular cell types. It is possible to evaluate stem cell differentiation and the development of desired cell types using methods like immunohistochemistry, gene expression analysis, or non-invasive imaging employing certain molecular markers. This aids in assessing the degree of functional recovery and tissue regeneration brought on by nanomaterial-conjugated stem cell treatment (Dong et al., 2021). By encouraging cell adhesion, motility, and interactions with the extracellular matrix, nanomaterials can improve stem cell engraftment and integration. The assessment of stem cell engraftment, distribution, and integration into the host tissue is made possible by monitoring techniques such molecular imaging or histological examination. This sheds light on how successful tissue regeneration and functional recovery have been.

Stem cell therapy can be combined with nanoparticles to further improve monitoring methods and take advantage of the special qualities of nanomaterials. For instance, imaging methods that make use of contrast agents or nano sensors based on nanomaterials can deliver high-resolution and real-time data on stem cell behavior and tissue integration. Additionally, direct monitoring of stem cell functionality and reactions within the host tissue may be made possible through the creation of biosensors based on nanomaterials (Vissers et al., 2019). Monitoring becomes even more important in the context of nanomaterial-conjugated stem cell therapy in order to optimize treatment plans and take advantage of the positive effects of nanomaterials in improving stem cell behavior and therapeutic results. The implementation of nanomaterial-conjugated stem cell therapies into clinical practice will be facilitated by ongoing developments in monitoring technology, opening up new directions for regenerative medicine and enhancing patient outcomes (Dong et al., 2021).

## 7 Challenges associated with stem cell therapy, nanomedicine, and clinical status

Despite the progress of stem-cell, important gaps remain and new questions continually arise. Stem-cell therapy faces several significant challenges, both ethical and practical, thus posing a hurdle in their transformation from bench to bedside. Ethical concerns are particularly pronounced with potential risk of tumorigenicity from undifferentiated hiPSCs and the destruction of human embryos for ESCs derivation, leading to widespread ethical debates and regulatory hurdles. While iPSCs offer a less controversial alternative by reprogramming adult cells, ethical issues surrounding genetic manipulation still persist. Additional challenges facing research in the utilization of ESCs are how to reduce contamination and cancer risks. Currently, immunological rejection is a major struggle, whereby transplanted stem cells, particularly those derived from donors, are being recognized as foreign and thus get rejected by the recipient’s immune system.

**FIGURE 11:**
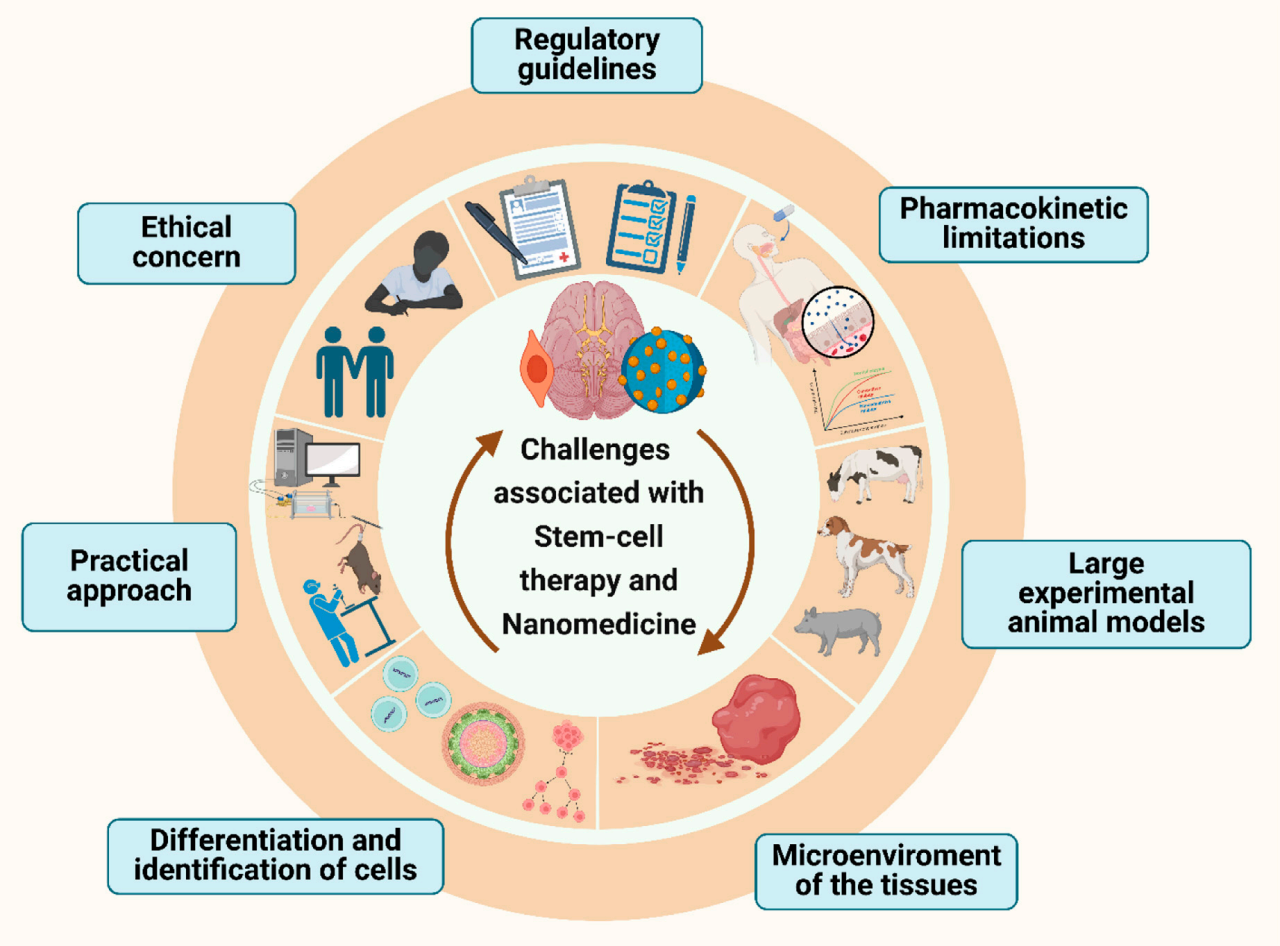
Summarization of key challenges associated with stem cell therapy and nanomedicine highlighting barriers such as clinical translation, ethical and practical conerns in the treatment of NDDs.

Lately, even though the term ‘translational’ has evolved to characterize a line of research inquiry that aimed at bridging the gap from bench to bedside and beyond, administering interventions outside of controlled clinical trials has endangered patients, jeopardized the integrity and trust of public in medical research, thus hindering the legitimate efforts to advance scientific knowledge. Numerous instances of litigation arise on the basis of misrepresentation or inappropriate informed consent yearly. Obtaining truly informed consent in SC-based trials in NDDs is challenging, involving barriers to disclosure and assessment of capacity for NDDs patients. A truly informed consent is valid when a prospective participant voluntarily consents for enrolment after receiving a thorough disclosure of the procedure which conveys highly complex scientific information, risks involved and any information that could affect patient’s health (Hellmers et al., 2016). Several studies have raised concerns about insufficient “standard” consent processes which threatens the independent regulatory review, thus minimizing respect for patient autonomy and transparency and eventually augmenting the effects of SCs therapeutic misconception and challenges (Hellmers et al., 2016). The latter is particularly a setback for people who have cognitive impairment thus needing surrogate decision making, which hence could be compromising. In recent years, hiPSC-based therapies have made significant strides, advancing into early clinical trials and demonstrated great potential in treating neuropathies including PD or MS through cell replacement presenting a critical strategy in addressing NDDs. However, it has also become a frequent target of scrutiny, owing to the risky interventions in humans and the questionable ethical practices and policies within the research and clinical framework that hampers its progression. Ethics of SCs is often questioned from a justice-based standpoint, whereby some argue that money are being spent on expensive and complex biomedical research but could rather be utilized for catering to public health for the underprivileged community (Ellison, 2016). Thus, ethical and legal issues arise in regard to possible health risks, permission given for nano-research and nano-medical technology availability for everybody. The complexity of the therapy affects the costs including stem cell injections, high-quality facilities, Additionally, the inadequacy of suitable stem cell types for cell replacement treatment in NDDs patients has hampered the progress of this promising therapeutic approach (Puranik et al., 2022). Although autologous stem cell transplants, using the patient’s own cells, can minimize the deficit of neurological diseases, the feasibility is compromised due to the limitations in cell availability and quality and intricate challenges encountered by scientists in identification of stem cells in adult tissues. Ensuring the precise differentiation of stem cells into the desired cell type is critical, yet difficult to achieve consistently. Mis-regulation can lead to the formation of unwanted cell types or even tumorigenesis. Additionally, delivering stem cells to the targeted area in the body and ensuring their survival and integration into existing tissues pose significant hurdles. The microenvironment in the diseased or damaged tissue can be hostile to newly introduced cells, affecting their viability and function. Furthermore, the long-term stability and functionality of transplanted stem cells remain uncertain, requiring extensive research and long-term clinical studies to understand their behavior fully. Research studies emphasize on the importance of nanomedicine and stem cell clinical applications in larger animals including swine, cattle, chimpanzees as they have been instrumental in advancing nanotechnology-based stem cell therapy, allowing a more realistic set of estimates of the quality and cost-effectiveness of new clinical trials. They play a significant role in establishing the safety of stem cell and nanotechnological applications since the dosages of biologics, the route of administration and therapy outcomes can be extrapolated readily to humans, compared to rodents’ usage. However, the attempts of larger animal use are limited for regenerative medicine owing to the lack of stable and well-characterized stem cell lines and protocols for their maintenance, differentiation and monitoring of cell status (Harding et al., 2013). In tackling NDDs where the challenges to elucidate the mechanistic regulation due to the complexity of human nervous system and differential degenerative progression is ongoing, finding larger model systems which may precisely mimics the disease condition and may appear to give a more robust recapitulation of human neurological disorders, can be challenging and expensive, thus hindering effective therapeutic translation (Eaton and Wishart, 2017; Yin et al., 2022). Nanomedicine also encounters several challenges. One of the foremost is the issue of toxicity. Nanoparticles, due to their small size and high reactivity, can exhibit toxic effects that are not present in their bulk counterparts. This toxicity can lead to unintended side effects and long-term health issues, complicating their approval and use in clinical settings. The biodistribution and accumulation of nanoparticles in the body are not fully understood, raising concerns about their potential to cause harm to organs or tissues. The production and standardization of nanoparticles present additional challenges. Manufacturing nanoparticles with consistent size, shape, and surface properties is technically demanding, yet crucial for ensuring predictable behavior in biological systems. Variations in these properties can lead to significant differences in how nanoparticles interact with cells and tissues, affecting their efficacy and safety. Regulatory challenges also abound in nanomedicine as summarized in Figure 11.

The unique properties of nanoparticles do not fit neatly within existing regulatory frameworks, necessitating the development of new guidelines and testing protocols to ensure their safety and efficacy. Delivery of nanoparticles to the targeted site within the body is another significant hurdle. Despite their promise as delivery vehicles, NPs can pose risks to human health by penetrating systemic circulation and accumulating in sensitive organs. Their physicochemical properties such as size, surface charge, and composition substantially affect biological interactions influencing pharmacokinetics, pharmacodynamics, and toxicity. These interactions may lead to oxidative stress, altered cellular uptake, or the release of toxic ions, thereby raising further concerns regarding their long-term safety (Feigin et al., 2020). While nanoparticles have the potential to cross biological barriers, including the blood-brain barrier, achieving targeted delivery in a controlled manner is complex. Off-target effects can reduce the efficacy of treatment and increase the risk of side effects. Additionally, the long-term fate of nanoparticles in the body and their potential to induce immune responses remain areas of active investigation. The interactive mechanism of nanomaterials and stem cells is still not well established. Major challenges involving how nanomaterials and nanostructures influence the stem cell replacement therapy and their metabolism. Generally, the NP technologies used to monitor *in-vivo* non-invasive stem cells must allow the long-term and sensitive position of cells to prevent cytotoxicity as much as possible. Generally, NP technologies which are often utilized to monitor *in-vivo* non-invasive stem cells in order to minimize cytotoxicity, need to permit the long-term and sensitive location of cells. Conversely, it is crucial to remember that hardly any NPs have been employed to track patients for therapeutic stem cells. This can be explained by the requirement that NPs undergo a critical phase of characterization for both chemical composition and biological effects on stem cells, including rate of viability following loading, the impact on stem cell migration, differentiation, encoding and the assessment of potential short- and long-term cytotoxicity, before they can be approved for therapeutic use. Table 2 details the clinical trials conducted or in trial to evaluate stem cell-based interventions in NDDs with study objectives and outcomes.

**TABLE 2:** Overview of clinical trials involving various stem cells and their clinical trials, highlighting the NDDs being studied.

Stem cells	Types	Clinical trial ID	Clinical trial phase	Condition/Disease	Purpose of study	Ongoing/Completed	References
Human Mesenchymal Stem cells (HMSC)	Adult stem cells	NCT04040348	Phase 1	AD	Testing the safety, possible side effects and effectiveness of MSCs infusion in mild to moderate AD patients	Completed	A Phase I (2019)
Autologous Bone Marrow derived stem cells (BMSC)	Pluripotent stem cells	NCT02795052	NA	PD ALS AD Dementia	Involve isolation of BMSC and transfer to vascular system to check neurologic functional improvement	Ongoing	ClinicalTrials.gov (2016)
Adipose-Tissue Derived Stem cells (ADSCs)	Pluripotent stem cells	NCT02383654	Phase 1	ALS	Study safety and effectiveness of ADSCs	Completed	ClinicalTrials.gov (2015)
HLA-haplo matched Allogenic Bone Marrow Derived Stem cells	Pluripotent stem cells	NCT01758510	Phase 1	ALS	Determine safety of these SCs through intrathecal delivery for ALS therapy	Completed	Corestemchemon Inc (2012)
Autologous Mesenchymal stem cells (MSCs)	Adult stem cells	NCT05167721	Phase 2	MSA	Assess optimal dosing frequency, effectiveness and safety of MSCs delivered into the spinal fluid	Ongoing	ClinicalTrials.gov (2021)
HESCs -derived Dopaminergic cells for PD	Embryonic stem cells	NCT05635409	Phase 1	PD	Assess the safety of STEM-PD product in patients	Ongoing	University et al. (2021)
Human amniotic epithelial stem cells (hAESCs)	Embryonic stem cells	NCT05691114	Phase 1	PD	Explore the safety, tolerability of hAESCs	Ongoing	Shanghai i CELL Biotechnology Co and China (2023)
Human amniotic epithelial stem cells (hAESCs)	Embryonic stem cells	NCT04414813	Early Phase 1	PD	Evaluate safety and efficacy of stereotactic transplantation of hAESCs	Completed	Shanghai iCELL Biotechnology Co and China (2019)
Allogenic Bone marrow derived mesenchymal stem cell (MSC)	Adult stem cells	NCT04506073	Phase 2	PD	Determine the safety of dosage for MSC infusions to slow down progression of disease	Completed	Research et al. (2020)
Mesenchymal Stem cells (MSC)	Adult stem cells	NCT03252535	Phase 2	HD	Dose-response evaluation of Cellavita HD	Completed	Ltda (2017)

Overcoming these challenges requires interdisciplinary collaboration and significant advances in both nanotechnology and biomedical sciences. However, since these challenges exist, there is a need for continued cooperation of pharmaceutical design and toxicology with clinical assessment of stem cells, cell therapy and nanomedicine.

## 8 Concluding remarks and future roadmap

Over the last few decades, the rapid advancement of nanotechnology, coupled with stem cell therapy has yielded promising therapeutics approaches in NDD’s treatment. This synergy appears to address certain limitations and challenges inherent in stem cell therapies. Stem cell nanotechnology has potentially facilitated deeper understanding of the intricate mechanism underlying NDDs, enabling mitigation characterized by enhanced safety, efficacy and precision. This joint venture of these two most prominent research domains is unlocking new possibilities for the production and study of SC therapy, thereby accelerating the potential clinical implementation of SCs in regenerative medicine. Even though this integration may face various challenges, similar to other novel interdisciplinary fields, nanotechnology is enhancing the recognition of SC-fate with the effective regenerative medicine research and clinical trials. Our review focuses on exploring stem cell classifications, the therapeutic potential of nanotechnological integration and their highly diversified potential pharmaceutical and therapeutic paradigm with future trends and perspectives. Hindered by the high order of information processing of human brain due to differences in neuronal circuits composition and integration of specialized neural networks in animals and humans, animal models are unable to precisely recapitulate this complexity, resulting in a lack of reliable and defined human neural tissues amenable to the dynamic functional assessment of neural circuits (Yan et al., 2024). Hence, AI is one immediate crucial tool to identify critical patterns and pathways involved in the earliest stage of the NDD’s pathogenesis (Marzi et al., 2023). As instance, the potential of AI as a means to comprehend the neural complexity of NDDs are highlighted, discussing the efficient evaluation and identification of patterns in large datasets including neuroimaging, neurological laboratory findings, psychological testing (Stefano, 2023). Artificial neural networks (ANN) are a cornerstone of AI which fundamentally contribute in predictive modelling, simulation of neurodegeneration and drug interactions and identification of potential targets thus holding great potential to revolutionize NDD’s management and ultimately improve patient outcomes and quality of life (Shao and Shen, 2023). Additionally, 3D bioprinting platform is engineered to assemble precise human neural tissues using commercial bioprinter. This technology assembling the neuronal and glial subtypes using bioprinting strategies including droplet-based and laser-based methods, aiming to mimic human neural tissues with 3D cytoarchitectures will likely be useful for understanding the wiring and mechanism of the neuronal network, modelling neuropathological processes and serving as new avenues for drug testing (Yan et al., 2024). The applicability of both stem cells and nanomedicine in the treatment of Neurodegenerative disorders is practically unbounded and is considered very promising mainly due to the current state of the situation that requires more significant advancement in therapy and a better outcome of patients with NDDs (Yan et al., 2024). Nanotechnology comes to play these roles in overcoming such challenges of delivering, differentiating and monitoring of stem cells besides being able to cross the blood-brain barrier (BBB) to directly administer therapeutic moieties to the CNS. Recent developments such as the use of nanoparticles as markers and stem cell delivery through the nasal cavity are steadily being accepted as better methodologies in the improvement of delivery of stem cell remedies. Nanocarriers that can serve multiple purposes are the target of modification with surfactants, surface functionalization like Polyethylene glycol (PEGylation) to suppress immune cross reactivity and hyper sensitivity) and specific targeting molecules can be used to deliver drugs and control the immune response in the NDDs (Ahmad et al., 2022). Moreover, the advance area known as nanomedicine can complement gene therapy to address the core issues of diseases such as Parkinson’s and Alzheimer’s, as compared to the specific paradigm of curative therapy. The advancement of nanomaterials in the form of quantum dots, dendrimers, and liposomes even raises the success and accuracy of these treatments by providing a controlled drug delivery system and monitoring the therapeutic process. However, there are still many unresolved issues, including the protection and optimal translation of these highly innovative devices directly into the clinical practice (Afzal et al., 2022). Future investigative studies should focus on integrating nanotechnology with stem cell therapy, with an aim of developing personal, efficient treatments, and these may possibly redefine the course of disease management of NDs, thus, improving the standard of living of individuals suffering from these diseases. In other words, the combination of stem cell therapy and nanomedicine means that there is a worthy cause in the fight against Neurodegenerative disorders with a hint of what the future of treatments will look like, that is enhanced and efficient (Dong et al., 2021). By harnessing the power of gene-editing techniques, particularly CRISPR–Cas systems, to endow stem cells with immune-evasive properties, researchers can fashion stem cells that circumvent the immune system’s recognition mechanisms. They can also incorporate fail-safe features to ensure that the cells can be eliminated in the event of unforeseen complications. Such ‘stealth’ cells could, in principle, underpin a wide range of cell-replacement therapies, and billions of dollars have been invested in this work over the past 5 years. Neurodegeneration has devastating sequelae with traditional pharmacological treatment. To date, stem cell nanotherapeutics has led to revolutionary outcomes in regenerative medicine with the potential of SC transplantation which increases daily with promising experimental results in animal models. Although much work has been conducted in some small-scale experiments, human trials must still be performed to assess the potential side effects. Stem cell transplantation involving nanoparticles and hydrogels appears likely to be a pivotal feature of future clinical strategies for NDDs therapy by replacing dysfunctional neurons, and affording neuroprotective and neurorestorative functions through accurate and more efficient drug delivery and regeneration therapies. Combining these two strategies enhance the therapeutic effects on NDDs, thus providing new insights and future promises for successful regenerative medicine (Wei et al., 2023). Although various hurdles remain, nanotherapeutic approach combined with stem cell therapy in neural replacement and regenerative therapies continue to deepen, aiming to tailor treatment to individual patients with NDDs, based on their genetic makeup, disease stage and unique clinical manifestations. The future of regenerative medicine is heading towards personalized therapeutics with precision drug delivery and the mating of these two specialties of science play a pivotal role in realizing this vision. Nanotechnology carries in its wake, the advances of efficient, precise drug delivery systems for both *in vitro* and *in vivo* genetic engineering of stem cells. Additionally, it heralds the advances in nanoscale systems like microarrays to study the gene expression of stem cells, facilitating the creation of dynamic three-dimensional nano-environment for the maintenance and differentiation of stem cells in various neurodegenerative disease models (Mansour et al., 2023). With nanotechnology paving a way for an advance comprehension of *in vivo* stem cell therapy and by enabling the simulation of these environment in cultures, this scientific fusion seems to hold significant promise for pioneering new vistas in stem cell research for neurodegenerative healthcare systems and improving patient outcomes on an unprecedented scale.
