# Correction: Harnessing nanotechnology for stem-cell therapies: revolutionizing neurodegenerative disorder treatments – a state-of-the-art update

**DOI:** 10.3389/fphar.2025.1674993

**Published:** 2025-08-07

**Authors:** Neevashini Chengebroyen, Anmol Seelan, Kamal Yoonus Thajudeen, Saad Ali Alshehri, Aritra Biswas, Israrahmed Adur, Vino Sundararajan, Sajitha Lulu Sudhakaran, Harpreet Singh

**Affiliations:** ^1^ Jeffrey Cheah School of Medicine and Health Sciences, Monash University Malaysia, Selangor, Malaysia; ^2^ Department of Biological Sciences, Sunandan Divatia School of Science, Narsee Monjee Institute of Management Studies (NMIMS), Mumbai, India; ^3^ Department of Pharmacognosy, College of Pharmacy, King Khalid University, Abha, Saudi Arabia; ^4^ Department of Biotechnology, UNESCO Regional Centre for Biotechnology, Government of India, Faridabad, Haryana, India; ^5^ Manipal Institute of Regenerative Medicine (MIRM), Manipal Academy of Higher Education (MAHE), Bangalore, Karnataka, India; ^6^ Integrative Multiomics Lab, School of Bio-Sciences and Technology, Vellore Institute of Technology, Vellore, Tamil Nadu, India; ^7^ School of Pharmaceutical Sciences (Faculty of Pharmacy), IFTM University, Moradabad, Uttar Pradesh, India

**Keywords:** nanomaterial-conjugated regenerative therapy, neurodegenerative disorders, nanomedicine, scaffold, neuroprotective-nanotechnology stem-cell therapy, nanotechnology

In the published article, there was an error. A correction has been made to the Acknowledgments. This previously stated:

“The authors would like to humbly acknowledge the management of Monash University (Malaysia Campus) and Jeffrey Cheah School of Medicine and Health Sciences (JCSMHS), supporting her overall academic and research pursuits. Authors would like to gratefully acknowledge Sagnik Nag, from JCSMHS MUM, for his continued intellectual support and motivation. The authors extend their appreciation to the Deanship of Research and Graduate Studies at King Khalid University for funding this work through Large Research Project under grant number RGP2/316/46.”

The corrected statement appears below:

“The authors extend their appreciation to the Deanship of Research and Graduate Studies at King Khalid University for funding this work through Large Research Project under grant number RGP2/316/46.”

The original version of this article has been updated.

